# Macrophages inhibit *Aspergillus fumigatus* germination and neutrophil-mediated fungal killing

**DOI:** 10.1371/journal.ppat.1007229

**Published:** 2018-08-02

**Authors:** Emily E. Rosowski, Nicholas Raffa, Benjamin P. Knox, Netta Golenberg, Nancy P. Keller, Anna Huttenlocher

**Affiliations:** 1 Department of Medical Microbiology and Immunology, University of Wisconsin-Madison, Madison, Wisconsin, United States of America; 2 Microbiology Doctoral Training Program, University of Wisconsin-Madison, Madison, Wisconsin, United States of America; 3 Graduate Program in Cellular and Molecular Biology, University of Wisconsin-Madison, Madison, Wisconsin, United States of America; 4 Department of Bacteriology, University of Wisconsin-Madison, Madison, Wisconsin, United States of America; 5 Department of Pediatrics, University of Wisconsin-Madison, Madison, Wisconsin, United States of America; Rutgers New Jersey Medical School, UNITED STATES

## Abstract

In immunocompromised individuals, *Aspergillus fumigatus* causes invasive fungal disease that is often difficult to treat. Exactly how immune mechanisms control *A*. *fumigatus* in immunocompetent individuals remains unclear. Here, we use transparent zebrafish larvae to visualize and quantify neutrophil and macrophage behaviors in response to different *A*. *fumigatus* strains. We find that macrophages form dense clusters around spores, establishing a protective niche for fungal survival. Macrophages exert these protective effects by inhibiting fungal germination, thereby inhibiting subsequent neutrophil recruitment and neutrophil-mediated killing. Germination directly drives fungal clearance as faster-growing CEA10-derived strains are killed better *in vivo* than slower-growing Af293-derived strains. Additionally, a CEA10 *pyrG*-deficient strain with impaired germination is cleared less effectively by neutrophils. Host inflammatory activation through Myd88 is required for killing of a CEA10-derived strain but not sufficient for killing of an Af293-derived strain, further demonstrating the role of fungal-intrinsic differences in the ability of a host to clear an infection. Altogether, we describe a new role for macrophages in the persistence of *A*. *fumigatus* and highlight the ability of different *A*. *fumigatus* strains to adopt diverse modes of virulence.

## Author summary

Immunocompromised patients are susceptible to invasive fungal infections, including aspergillosis. However, healthy humans inhale spores of the fungus *Aspergillus fumigatus* from the environment every day without becoming sick, and how the immune system clears this infection is still obscure. Additionally, there are many different strains of *A*. *fumigatus*, and whether the pathogenesis of these different strains varies is also largely unknown. To investigate these questions, we infected larval zebrafish with *A*. *fumigatus* spores derived from two genetically diverse strains. Larval zebrafish allow for visualization of fungal growth and innate immune cell behavior in live, intact animals. We find that differences in the rate of growth between strains directly affect fungal persistence. In both wild-type and macrophage-deficient zebrafish larvae, a fast-germinating strain is actually cleared better than a slow-germinating strain. This fungal killing is driven primarily by neutrophils while macrophages promote fungal persistence by inhibiting spore germination. Our experiments underline different mechanisms of virulence that pathogens can utilize—rapid growth versus dormancy and persistence—and inform future strategies for fighting fungal infections in susceptible immunocompromised patients.

## Introduction

Humans inhale hundreds of *Aspergillus fumigatus* spores from the environment every day and yet almost all immunocompetent individuals successfully contend with the fungal infection. Immunocompromised patients, however, especially acute leukemia patients, hematopoietic cell transplant recipients, and solid-organ transplant recipients, are at risk of developing invasive aspergillosis [1]. In invasive disease, *Aspergillus* spores germinate into filamentous hyphae and invade and destroy tissues and organs, with mortality rates as high as 50 to 60% in patient populations [1]. Limited antifungal treatments exist and there is growing resistance among fungi to these drugs [2]. The development of successful immunotherapy-based treatments to this infection requires a more comprehensive understanding of the interplay of immune mechanisms that control *Aspergillus in vivo*.

In immunocompetent individuals, the innate immune system is generally sufficient for *A*. *fumigatus* clearance, with both macrophages and neutrophils playing major roles [3]. Macrophages primarily target and phagocytose conidia (spores), and can kill conidia *in vitro* within hours [4, 5]. *In vivo*, however, the requirement for and role of macrophages is unclear, with some studies reporting no effect on survival in macrophage-depleted mice [6] and others demonstrating an increased fungal burden in such mice [7]. Neutrophils, on the other hand, are consistently required in mice in response to *Aspergillus* [6, 8], and neutropenia is a key risk factor for patients in the development of invasive aspergillosis [9]. Neutrophils can kill conidia *in vivo* [10] and inhibit conidial germination both *in vitro* and *in vivo* [11, 12]. Immune cell populations can also influence the activity of other immune cell types, and CCR2+ inflammatory monocytes can increase neutrophil function in response to *Aspergillus* through pro-inflammatory gene expression [13, 14]. However, the full extent of macrophage/monocyte and neutrophil interactions throughout the course of infection, and their effect on fungal growth and clearance, are not known.

Perhaps the most important step in *Aspergillus* pathogenesis is germination, the developmental switch from resting, dormant conidia to filamentous, invasive growth [15]. These different fungal forms induce differential activation of immune cells *in vitro* and recruitment of these cells *in vivo* [16–18]. After invasive hyphae have developed, neutrophils can respond with a variety of killing mechanisms [11, 19–21]. However, how this switch, and specifically earlier stages of fungal germination prior to the development of extensive invasive growth, affects immune activation, fungal clearance, and disease progression *in vivo* is largely unknown.

Larval zebrafish provide an ideal model in which to study the early effects of fungal germination as well as the interplay between multiple immune cell types throughout infection. Larval zebrafish are transparent and can be non-invasively imaged and therefore allow for visualization of innate immune cell behavior and fungal development in a live, intact host throughout a multi-day infection. While murine models require enormous fungal inoculums (~10^7^ conidia) and host immunosuppression in order to directly visualize immune cell-pathogen encounters within the context of host tissue, the larval zebrafish allows for visualization of smaller doses (10–100 conidia) in wild-type hosts [22]. *Aspergillus* infection models in zebrafish have already been developed by our lab and others and recapitulate many aspects of human and mouse infection, including initial phagocytosis of conidia by macrophages and post-germination neutrophil recruitment [21, 23]. These models have already provided new insight into the biology of immune responses to *Aspergillus* such as the observation of spore transfer between macrophages [24].

In this study, we have infected larval zebrafish with *Aspergillus fumigatus* strains derived from patient isolates Af293 and CEA10. Heterogeneity among *A*. *fumigatus* isolates has been increasingly recognized [25], and Af293 and CEA10 in particular are known to be differentially virulent in specific mouse and zebrafish models [26–28]. The genomes of these strains differ by ~50,000 single nucleotide polymorphisms (SNPs), which is an approximately average level of genetic diversity among sequenced *A*. *fumigatus* isolates [28]. Af293 and CEA10 strains also possess differences in growth [27], activation of the host immune response [27, 29], response to hypoxia [26], secondary metabolism [30, 31], and response to light [32]. We aimed to use these two different strains coupled with live imaging in larval zebrafish to identify and characterize differential neutrophil and macrophage behaviors in response to *A*. *fumigatus* infection.

We report that while CEA10 is generally regarded as more virulent than Af293 [26, 27], CEA10-derived strains are killed better than Af293-derived strains *in vivo*, even while CEA10-derived strains germinate faster. Host inflammatory activation, partially through Myd88, is required for clearance of a CEA10-derived strain, but strikingly, is not sufficient for clearance of an Af293-derived strain. Macrophages only marginally contribute to clearance of CEA10-derived strains. Instead, macrophages primarily provide a protective niche for spores, forming tight, dense clusters around the fungus, inhibiting spore germination. Our data demonstrate that germination actually drives fungal clearance through neutrophil-mediated killing.

## Results

### A CEA10-derived strain of *Aspergillus fumigatus* is killed better than an Af293-derived strain *in vivo*

Differences in the virulence of two common lab strains isolated from patients, Af293 and CEA10, have been reported, but the mechanisms underlying these differences remain incompletely understood [26–28]. In particular, the ability of wild-type hosts to contend with and clear these two different infections is not known. To investigate such clearance, we decided to apply a published method to visually quantify *Aspergillus* spore killing [10], to our established model of *Aspergillus* infection in transparent zebrafish larvae [21], giving us the ability to visualize fungal killing in a live, intact host. This live-dead staining method utilizes *Aspergillus* strains expressing a fluorescent protein (in these experiments, YFP or GFP) and coated in an AlexaFluor molecule of a different color (here, AlexaFluor594). Live spores are visualized as YFP/GFP signal surrounded by AlexaFluor signal, while killed spores appear as AlexaFluor signal only. We used strains derived from Af293 and CEA10 that express YFP and GFP, tagged to cytosolic proteins. Throughout this paper we will refer to Af293-derived and CEA10-derived strains in the text and figures as simply Af293 and CEA10, respectively, for ease of discussion, but we list actual strains used for each experiment in figure legends and the details of all strains in Methods.

**Fig 1:**
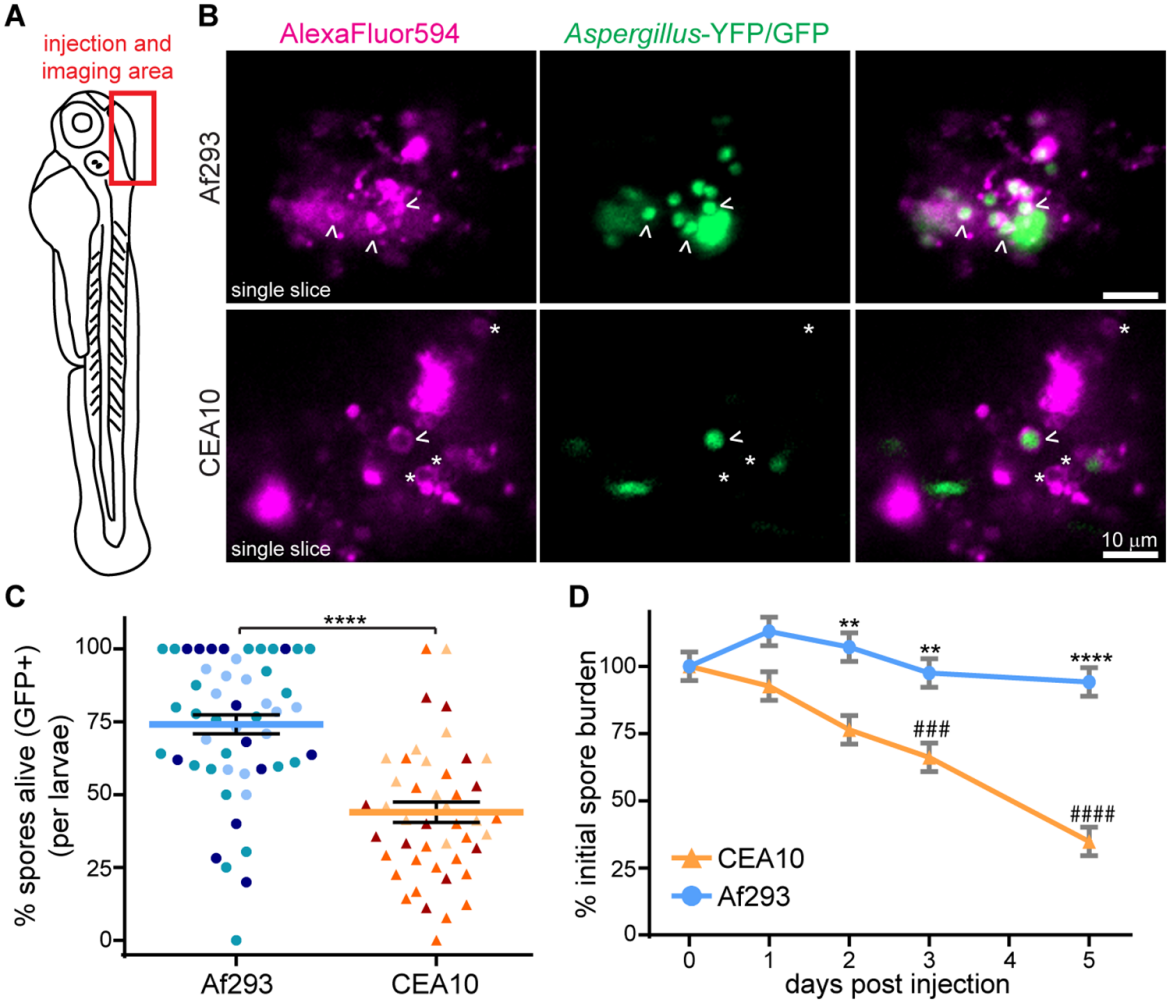
A CEA10-derived strain is killed more than an Af293-derived strain *in vivo*. **A**. Schematic of 2 dpf zebrafish larvae. Area of *Aspergillus* spore injection (hindbrain) and imaging is indicated. **B, C**. TBK1.1 (Af293) or TFYL49.1 (CEA10) spores express YFP or GFP and have AlexaFluor594 conjugated to the spore cell wall. 2 dpi larvae were imaged and the percentage of spores killed (GFP+/total) was quantified. Representative images of single z slices (B) and quantification over three experiments (C) are shown. Arrowheads indicate alive spores, asterisks indicate killed spores. Scale bar represents 10 μm. Each symbol represents one larvae, color-coded by experiment. Lines represent lsmeans ± SEM. Af293 n = 51, CEA10 n = 45. **D**. Fungal burden was monitored by CFU platings from single homogenized larvae infected with TBK1.1 (Af293) or TFYL49.1 (CEA10). CFUs from 24 larvae (3 replicates, 8 larvae each) per condition per day were measured. Average injection CFUs: Af293 = 150, CEA10 = 140. For all analyses, data represent lsmeans ± SEM from three pooled experiments, P values calculated by ANOVA. Asterisks represent significance between strains, # represent significance compared to day 0. See also S1 Fig.

We injected dual-labeled spores into the hindbrain of wild-type 2 day post fertilization (dpf) zebrafish larvae and imaged this hindbrain region; all images presented throughout this study are oriented as indicated (Fig 1A). Confocal imaging of these larvae 2 days post injection (dpi) revealed that CEA10 is killed significantly better than Af293, with a higher percentage of dual-labeled AlexaFluor594^+^, YFP/GFP^+^ spores in an Af293 infection compared to a CEA10 infection (Fig 1B and 1C). These results were surprising to us given that CEA10 has been reported to be more virulent than Af293 in some immunosuppressed mouse models of infection [26, 27] as well as in data from our lab in neutrophil-defective zebrafish larvae [28]. To confirm this difference in fungal killing, we quantified colony-forming units (CFUs) from homogenized single larvae over the course of a 5 day infection. This experiment recapitulated the finding that fungal burden of this CEA10 strain is controlled significantly better by larval zebrafish than the Af293 strain (Fig 1D). While Af293 persists in larvae throughout the course of infection, CEA10 is slowly cleared, so that by 2 dpi, the fungal burden in CEA10-infected larvae is significantly lower than that of Af293-infected larvae. Additionally, by 3 dpi, CEA10 fungal burden is significantly less than the initial dose. Af293 is never significantly cleared compared to the initial burden under the experimental conditions. We also confirmed that this trend holds for multiple Af293- and CEA10-derived strains, as well as the parental strains (S1 Fig).

### Myd88-mediated inflammatory activation is required for clearance of a CEA10-derived strain

**Fig 2:**
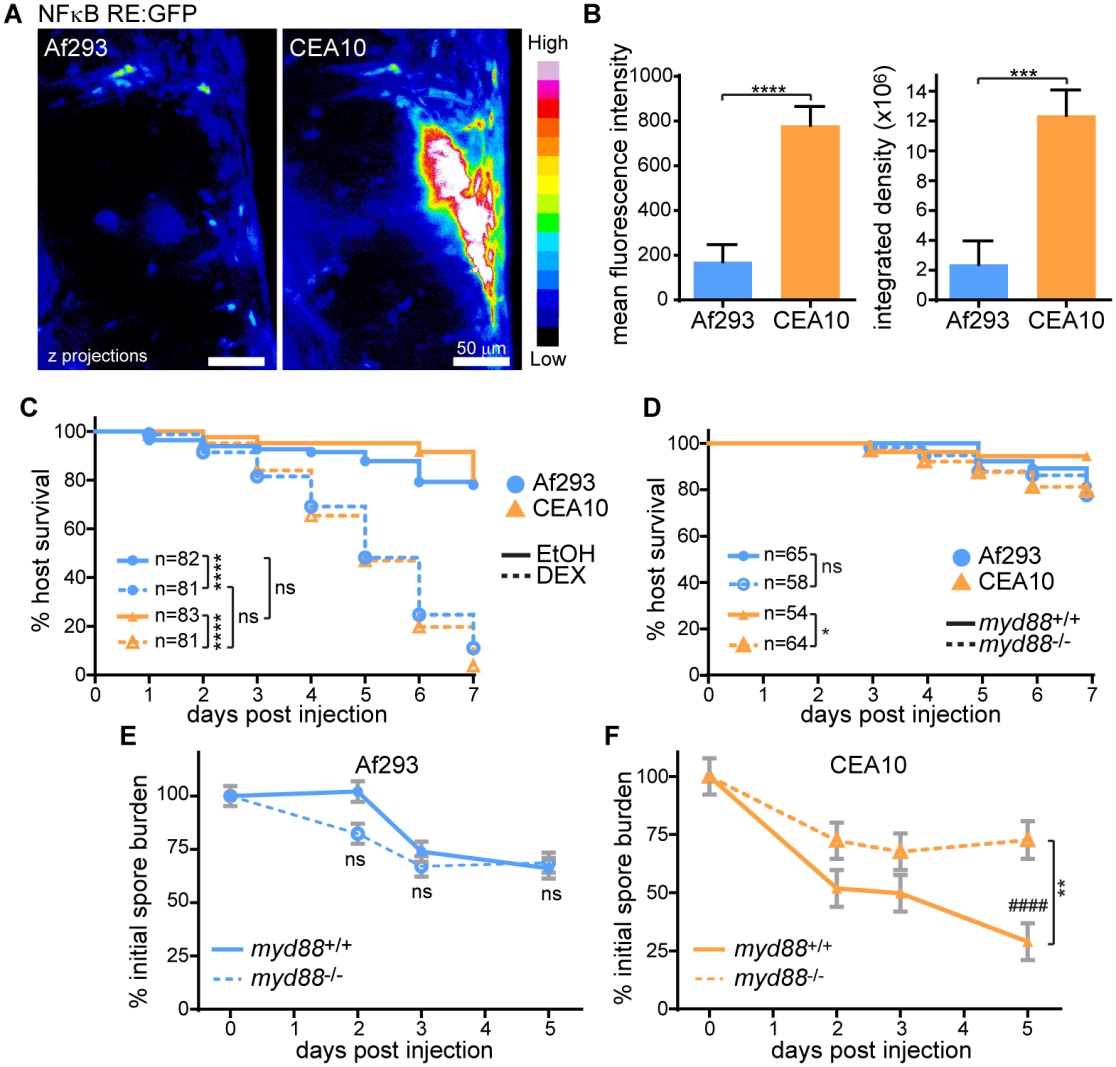
Inflammatory activation is required for clearance of a CEA10-derived strain. **A, B**. NF-κB RE:EGFP larvae were infected with non-fluorescent TJW55.2 (Af293) or CEA17 KU80Δ (CEA10) spores and imaged 2 dpi. Representative images (A) and quantification from three pooled experiments (B) are shown. Scale bar represents 50 μm. CEA10 n = 22; Af293 n = 26. **C**. Larvae were treated with dexamethasone (DEX) or ethanol (EtOH) vehicle control directly after infection with TFYL81.5 (Af293) or TFYL49.1 (CEA10), and larval survival was monitored. Average injection CFUs: Af293 = 34, CEA10 = 33. **D-F**. *myd88*^-/-^ or control larvae were infected with TBK1.1 (Af293) or TFYL49.1 (CEA10) and larval survival and/or fungal burden was monitored. Fungal burden was determined by CFU platings from single homogenized larvae. Asterisks represent significance between *myd88*^-/-^ and control larvae, # represent significance compared to day 0. Average injection CFUs: D, F: Af293 = 27, CEA10 = 32; E: Af293 = 66. For all analyses, data represent 3 pooled replicates. For CFU experiments, CFUs from 24 larvae (8 larvae per replicate) per condition per day were measured. Intensity values and CFU analyses represent lsmeans ± SEM from three pooled experiments, P values calculated by ANOVA. P values for survival analyses calculated by Cox proportional hazard regression analysis. See also S2 Fig.

Compared to Af293, CEA10 differentially activates specific inflammatory pathways in mice [27], and we first hypothesized that the differential killing of CEA10 was due to greater inflammatory activation. We utilized an *NF-κB RE*:*EGFP* reporter line [33] to measure differences in NF-κB activation after infection with Af293- or CEA10-derived non-fluorescent strains in larval zebrafish. At 2 dpi, CEA10-infected larvae have significantly more GFP signal and therefore NF-κB activation than Af293-infected larvae at the infection site, indicating that NF-κB activation is correlated with fungal killing (Fig 2A and 2B).

To determine whether general immune activation is required for fungal killing, we treated larvae with the corticosteroid dexamethasone immediately after spore injection. Neither Af293 nor CEA10 caused much death in control larvae, consistent with previous results in our zebrafish model and the idea that wild-type hosts can largely control *Aspergillus* infection [21]. Treatment with dexamethasone, however, caused both Af293- and CEA10-infected larvae to succumb rapidly and equally to infection (Fig 2C), consistent with the identical virulence of these two strains observed in a chemotherapeutic mouse model [26]. Control PBS-injected larvae treated with dexamethasone had minimal death (S2A Fig). Similar results were obtained after treatment with withaferin A, a non-specific inhibitor of NF-κB (S2B Fig). These results suggest that while CEA10 infection induces significantly more activation of NF-κB, under general immunosuppressive conditions, neither of these strains can be controlled by the host.

Both dexamethasone and withaferin A have broad effects, therefore we next investigated the role of specific pathways upstream of NF-κB in fungal killing. Myd88 is an adaptor molecule downstream of TLR/IL-1R signaling that has previously been implicated in the response to *Aspergillus* [34, 35]. *myd88*^-/-^ larvae are more susceptible to CEA10, but not Af293, infection compared to wild-type controls (Fig 2D), consistent with the idea that Myd88-mediated immune activation is required specifically for the response to CEA10. This increase in susceptibility to a CEA10 strain of *myd88*^-/-^ larvae coincided with a significantly decreased ability to clear CEA10 fungal burden, while loss of Myd88 had no effect on Af293 fungal burden (Fig 2E and 2F). Knockdown of *myd88* did not inhibit activation of the *NF-κB RE*:*EGFP* reporter line by a CEA10-derived strain (S2C and S2D Fig). This is could be due to 1) other pathways that also activate NF-κB and/or 2) increased fungal growth in these larvae resulting in more inflammation. Together, these results show that CEA10 induces a more robust inflammatory response than Af293 and Myd88-dependent signaling is required for clearance of CEA10 fungal burden.

### CEA10-induced inflammatory activation is not sufficient for killing of Af293

**Fig 3:**
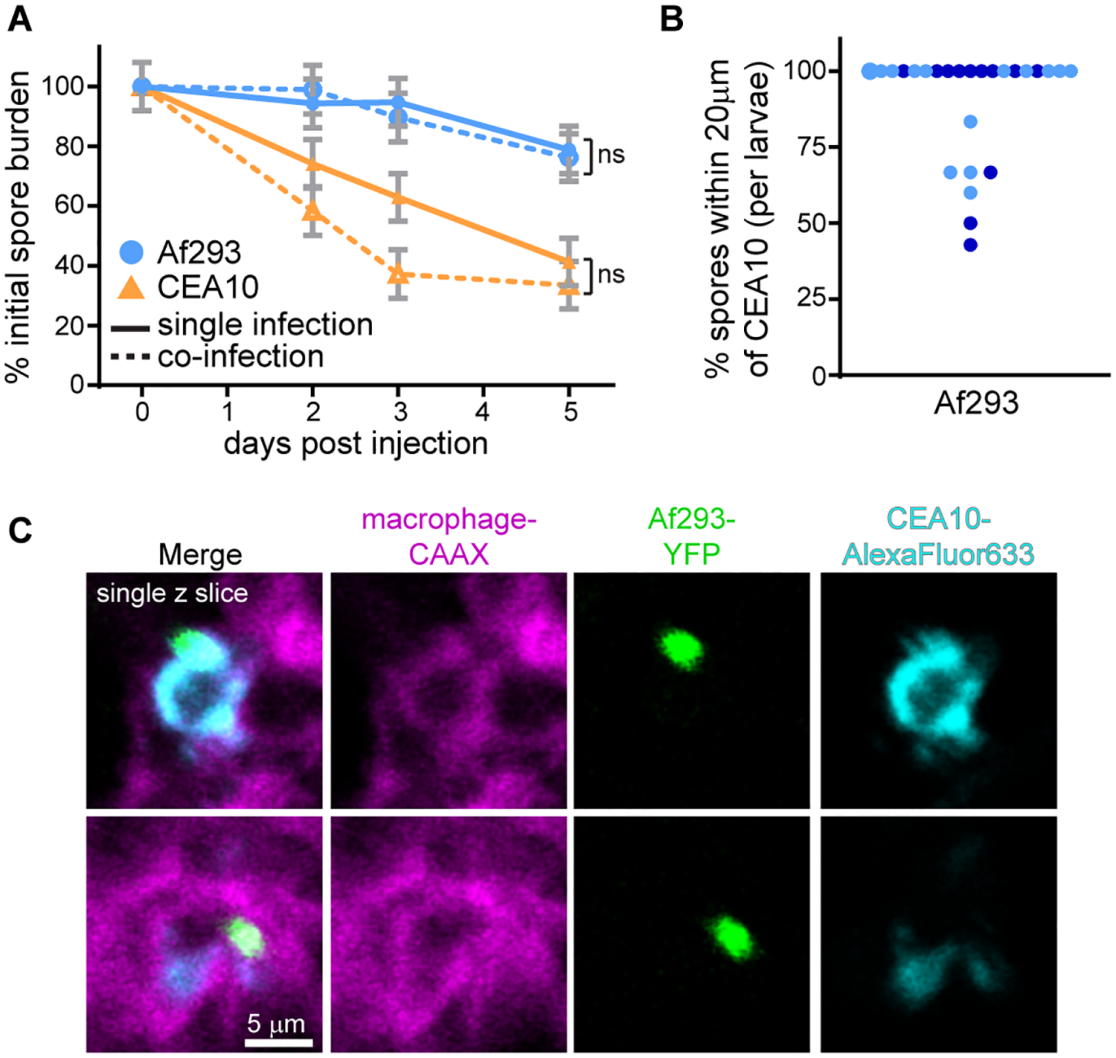
CEA10-induced inflammatory activation is not sufficient for killing of an Af293-derived strain. **A**. Fungal burden was monitored by CFU platings from single homogenized larvae infected either singly with TFYL49.1 (CEA10) or TBK5.1 (Af293) or co-infected with both strains. Average injection CFUs: Af293 = 53, CEA10 = 47, co-infection = 21 Af293 + 27 CEA10. CFUs from 24 larvae (8 larvae per replicate) per condition per day were measured; lsmeans ± SEM are shown from three pooled experiments, P values calculated by ANOVA. **B, C**. Macrophage-membrane labeled larvae (*mfap4*:*tomato-CAAX*) were co-infected with YFP-expressing TBK1.1 (Af293) and AlexaFluor633-labeled CEA17 KU80Δ (CEA10) and imaged 1 dpi. Percentage of Af293 spores within 20 μm of a CEA10 spore in each larvae from two replicates was quantified (B). Each point represents one larvae, color-coded by replicate. Single z-slice images from a representative larvae (C) are shown. Scale bar represents 5 μm. See also S3 Fig.

To specifically test whether the increased immune activation induced by this CEA10 infection is sufficient for fungal killing, we co-infected larvae with a red fluorescent Af293-derived strain and a green fluorescent CEA10-derived strain and performed CFU counts throughout a 5 day infection. As shown previously, in single infections this CEA10 strain is cleared over the course of infection while the Af293 strain persists (Figs 1D and 3A). In a co-infection, this same pattern remained—Af293 was not significantly cleared, even in the presence of an immune-activating CEA10 strain (Fig 3A). Imaging experiments confirmed that these spores can be found in the same or neighboring cells (Figs 3B, 3C and S3), suggesting that these killing differences are not due to local variation in immune activation, but instead to fungal-intrinsic phenotypes.

### Macrophages form tight, persistent, and dynamic clusters around *A*. *fumigatus*

**Fig 4:**
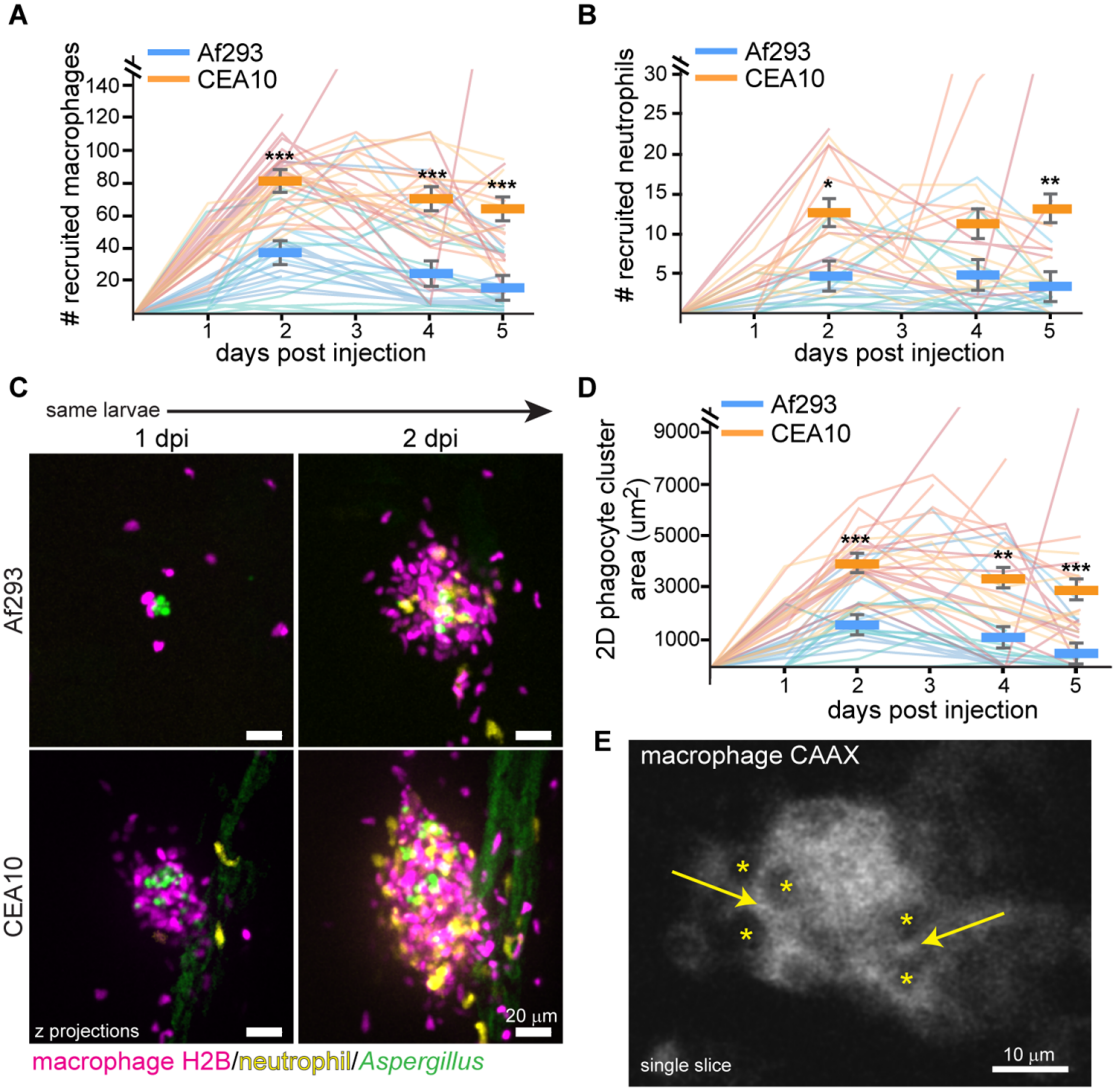
Macrophages form tight clusters around *A*. *fumigatus*. **A-D**. Dual macrophage-nuclear (*mpeg1*:*mcherry-H2B*) and neutrophil (*lyz*:*BFP*) labeled larvae were infected with YFP- or GFP-expressing *A*. *fumigatus* TBK1.1 (Af293) or TFYL49.1 (CEA10) strains and imaged days 1–5 post injection. Macrophage (A) and neutrophil (B) recruitment as well as phagocyte cluster size (D) were quantified. Representative images (C, scale bar represents 20 μm) and pooled quantification from 3 experiments (A, B, D) are shown. Each line in graphs represents one larvae followed for the entire course of infection, lines are color-coded by replicate, bars represent pooled lsmeans ± SEM, P values calculated by ANOVA. Af293 n = 25, CEA10 n = 27 for macrophage and cluster area quantification; Af293 n = 17, CEA10 n = 18 for neutrophil quantification. **E**. Macrophage-membrane (*mfap4*:*tomato-CAAX*) labeled larvae were infected with CEA17 KU80Δ (CEA10) and imaged 3 dpi. Asterisks represent cell nuclei, arrows indicate cell-cell junctions. Scale bar represents 10 μm. See also S4 Fig.

To further determine the mechanism by which this CEA10 strain of *A*. *fumigatus* is killed, we utilized the multi-day imaging capabilities of larval zebrafish to monitor the recruitment and behavior of macrophages and neutrophils in individual larvae over the course of a five day infection. By following individual larvae with both labeled macrophage nuclei (*mpeg1*:*mcherry-H2B*) and neutrophils (*lyz*:*BFP*) throughout a 5 day infection, we aimed to determine both differences in immune cell recruitment within larvae over time and population-level differences between larvae infected with a CEA10-derived strain versus an Af293-derived strain. On a population level, CEA10 infection recruited more macrophages and neutrophils than Af293 infection, throughout the entire time course (Fig 4A and 4B). These differences were significant as early as 2 dpi and up to 5 dpi (Fig 4A and 4B). However, the number of macrophages and neutrophils present at the site of infection was also dynamic, fluctuating from day to day in individual larvae (Fig 4A and 4B). In general, immune cell recruitment peaked at 2 dpi, decreasing slowly afterwards, coinciding with the timeline of fungal clearance (Fig 1D).

During this imaging, we were surprised to find that these recruited phagocytes form tight clusters around the injected *A*. *fumigatus*, as early as 1 dpi, that persist throughout the five day infection (Figs 4C, 4D and S4A, S1 Movie). These clusters were primarily made up of macrophages, but neutrophils also infiltrated, generally arriving after macrophages (Figs 4C and S4A). These macrophages form tight associations, with their membranes directly opposed and without space between the cells (Fig 4E). Germination of *A*. *fumigatus* spores inside the clusters could be found (S4A Fig). We also observed the apical extrusion of a cluster out of the hindbrain of two larvae, suggesting extrusion is a possible but rare mechanism for the host to eliminate the infection (S4B Fig). Quantification of phagocyte cluster size demonstrates that significantly larger clusters form in response to CEA10 compared to Af293, consistent with differences in observed numbers of phagocytes recruited (Fig 4C and 4D). Additionally, the size of these clusters is dynamic, changing in individual larvae throughout the course of infection, but also peaking at 2 dpi and decreasing in size as the infection is cleared (Fig 4C and 4D).

### A CEA10-derived strain is more virulent than an Af293-derived strain in a neutrophil-defective host

**Fig 5:**
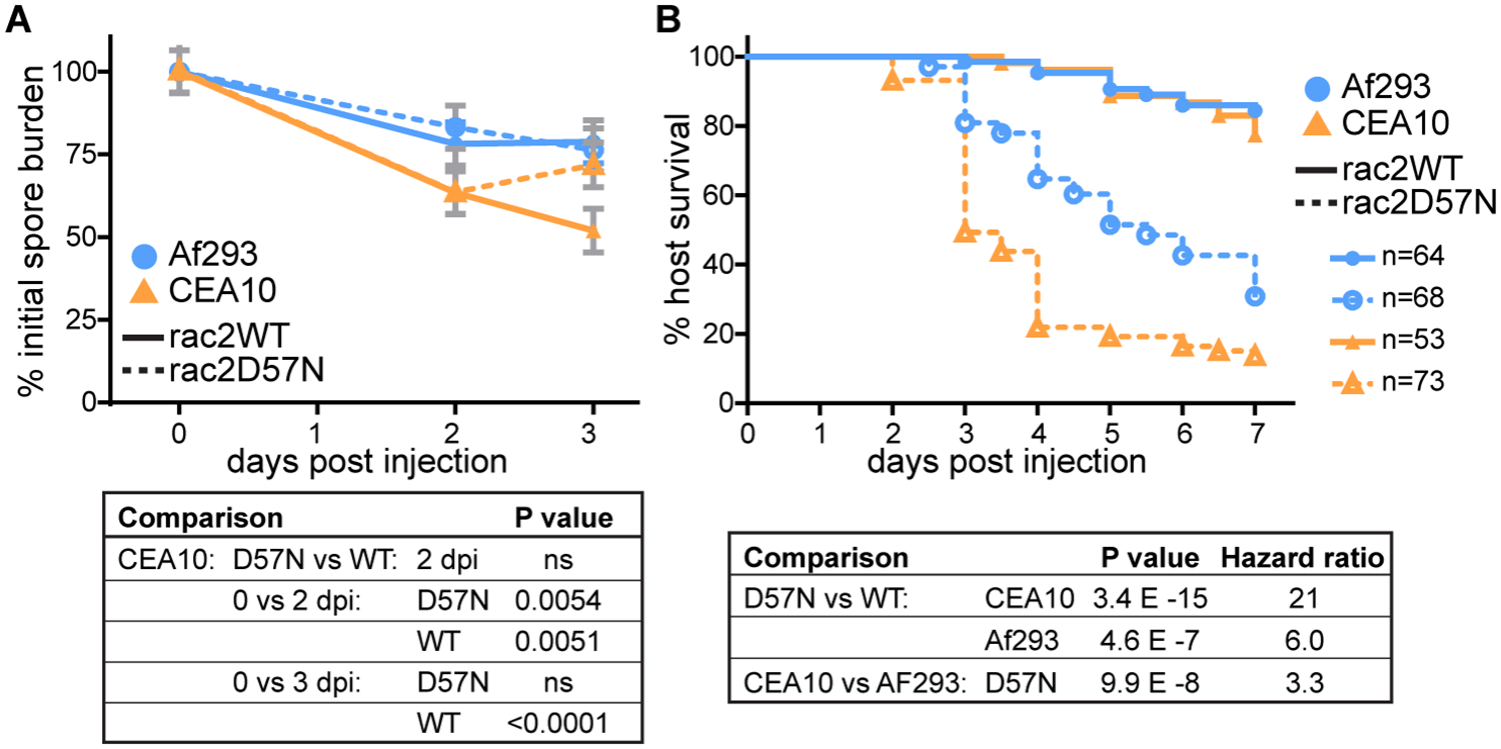
A CEA10-derived strain is more virulent in a neutrophil-defective host. Neutrophil-defective (*mpx*:*rac2D57N*) or control (*mpx*:*rac2WT*) larvae were infected with TBK1.1 (Af293) or TFYL49.1 (CEA10). **A**. CFUs were monitored, average injection CFUs: Af293 = 36, CEA10 = 32. **B**. Larval survival was monitored, average injection CFUs: Af293 = 35, CEA10 = 27. For all analyses, data shown are from 3 pooled replicates. For CFU analysis, data represent lsmeans ± SEM, P values calculated by ANOVA. CFU data are from 24 larvae (3 replicates, 8 larvae each) per condition per day. For survival analysis, P values were calculated by Cox proportional hazard regression analysis. See also S5 Fig.

Since macrophages are the predominant cell type infiltrating the site of infection and forming clusters around the fungus, we hypothesized that macrophages contribute to killing of CEA10-derived strains of *A*. *fumigatus*. Macrophages and neutrophils are the predominant immune cell types present in zebrafish larvae at this stage of development [36] and therefore, to determine the ability of macrophages alone to kill *A*. *fumigatus in vivo*, we performed fungal CFU counts after infection of a neutrophil-defective host (*mpx*:*rac2D57N*). These larvae express a dominant-negative copy of Rac2 under a neutrophil-specific promoter, preventing the migration of neutrophils out of the vasculature [37]. Consistent with previous observations (Fig 1), an Af293-derived strain was not significantly cleared in either neutrophil-defective or control hosts (Fig 5A). However, 2 dpi, neutrophil-defective larvae are able to decrease fungal burden of a CEA10-derived strain equivalently to the control, to ~60% of the initial dose (Fig 5A), indicating that early in infection macrophages have a low-level killing ability against CEA10. However, at 3 dpi, CFUs from these neutrophil-defective larvae increase, demonstrating that macrophages alone cannot contain the growth of CEA10 (Fig 5A). Because these larvae have fewer functional immune cells in total, we also tested a lower dose of CEA10, but found that the infectious dose did not have any effect on percent of fungal clearance (S5 Fig).

Over 80% of these neutrophil-defective larvae succumb to infection with CEA10 (Fig 5B). To compare the relative likelihood of neutrophil-defective larvae to succumb to CEA10 versus Af293 infection, we calculated hazard ratios, which represent the relative instantaneous risk of death throughout the experiment between two conditions. This calculation revealed that while neutrophil-defective larvae are significantly more susceptible to both Af293 and CEA10 infections than control larvae, these hosts are ~3 times more likely to succumb to CEA10 infection than Af293 infection (Fig 5B), consistent with our previous results in this zebrafish line [28]. So, while CEA10 is cleared significantly more than Af293 in wild-type larvae, it is also more virulent in a neutrophil-defective host. Altogether, these data suggest that macrophages do not account for the majority of fungal killing, instead implicating neutrophils.

### Macrophages provide a protective niche for CEA10

**Fig 6:**
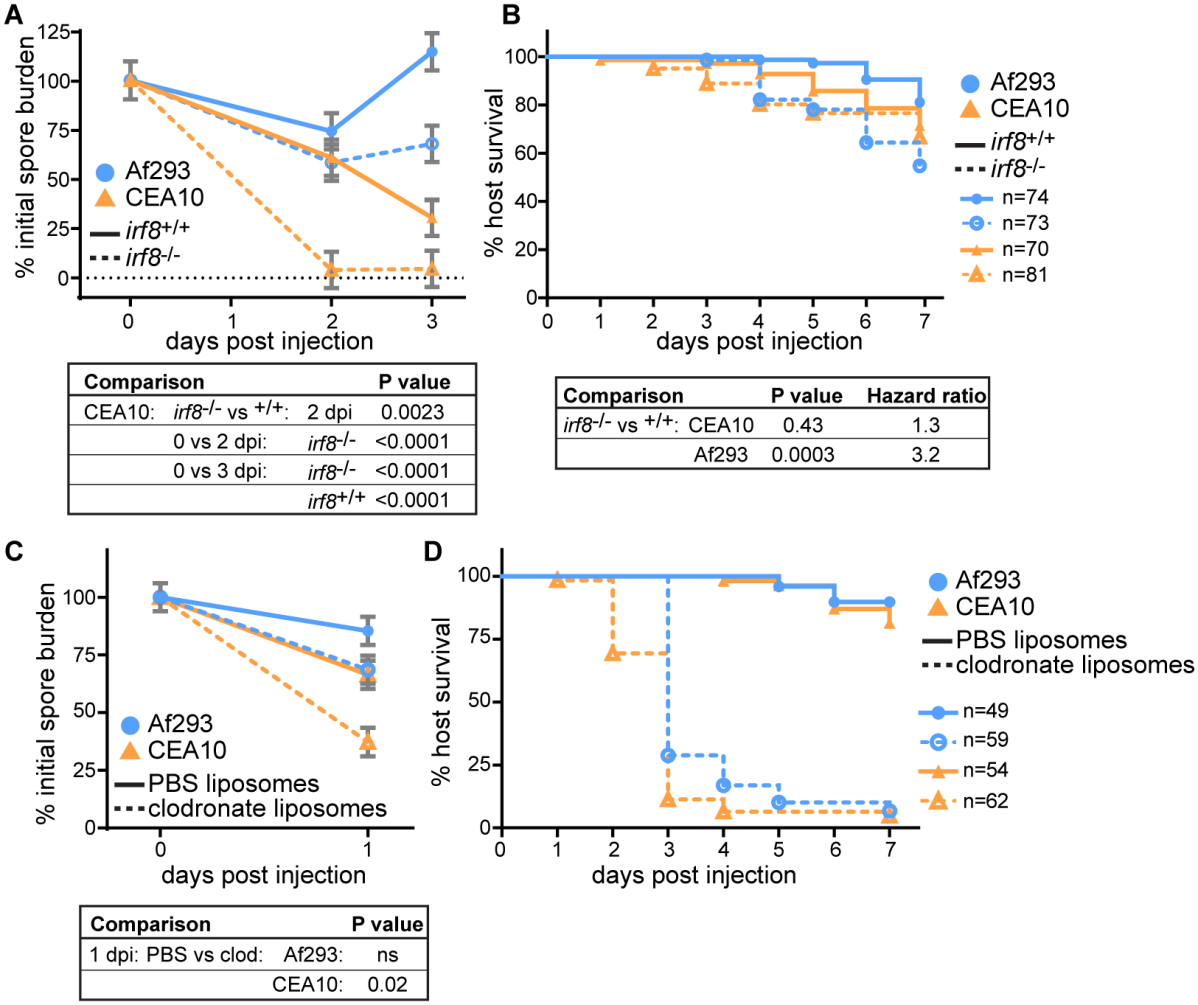
A CEA10-derived strain is cleared more efficiently by macrophage-deficient hosts than wild-type hosts. **A, B**. Macrophage-deficient (*irf8*^-/-^) or control (*irf8*^+/+^) larvae were infected with TBK1.1 (Af293) or TFYL49.1 (CEA10) and CFUs (A) or larval survival (B) were monitored. For CFU experiments, average injection CFUs: Af293 = 23, CEA10 = 23. For survival experiments, average injection CFUs: Af293 = 44, CEA10 = 31. **C, D**. Macrophage-depleted (clodronate liposomes) or control (PBS liposomes) larvae were infected with TBK1.1 (Af293) or TFYL49.1 (CEA10) and CFUs (C) or larval survival (D) were monitored. Average injection CFUs: Af293 = 37, CEA10 = 40. For all analyses, data shown are from 3 pooled replicates. For CFU analyses, data represent lsmeans ± SEM, P values calculated by ANOVA. All CFU data are from 24 larvae (3 replicates, 8 larvae each) per condition per day. For survival analyses, P values were calculated by Cox proportional hazard regression analysis. See also S6 Fig.

To specifically investigate the function of neutrophils in the response to these two *A*. *fumigatus* strains, we next infected macrophage-deficient larvae (*irf8*^-/-^). These zebrafish lack the transcription factor Irf8, and do not develop macrophages for the first 7 days after fertilization [38]. We again enumerated CFUs after infection with either an Af293-derived or a CEA10-derived strain. Remarkably, these macrophage-deficient larvae are able to clear CEA10 significantly more than control larvae (Fig 6A). While, again, wild-type hosts significantly decrease CEA10 fungal burden by 3 dpi, at 2 dpi almost 100% of injected CEA10 is cleared in macrophage-deficient hosts (Fig 6A). Consequently, when we monitored survival, CEA10-infected macrophage-deficient larvae succumbed to infection at an almost identical rate as wild-type controls (Fig 6B). Af293, on the other hand, was not significantly cleared in macrophage-deficient larvae (Fig 6A) and Af293 infection did result in significantly more death in macrophage-deficient larvae compared to controls (Fig 6B), consistent with previous results in zebrafish with this strain [21].

As a consequence of Irf8 deficiency, these larvae also develop a larger number of neutrophils [38]. However, larvae injected with clodronate liposomes also cleared a CEA10-derived strain, but not an Af293-derived strain, significantly better than control PBS liposome-injected larvae at 1 dpi (Fig 6C). As clodronate liposome injection specifically depletes macrophages without affecting neutrophils (S6A Fig), this result suggests that this increased early clearance is a specific consequence of macrophage depletion. Clodronate liposome-injected larvae do however succumb to infection (Figs 6D and S6B), indicating either that a larger number of neutrophils is required for complete fungal clearance and host survival in the absence of macrophages, and/or that clodronate liposomes affect neutrophil function without altering neutrophil numbers. These data demonstrate that the presence of macrophages actually protects injected CEA10 spores, but not Af293 spores, from neutrophil-mediated killing at early time points after infection, and suggest that macrophages might provide a protective niche for survival of certain fungal strains.

### CEA10 recruits more neutrophils than Af293 in macrophage-deficient hosts

**Fig 7:**
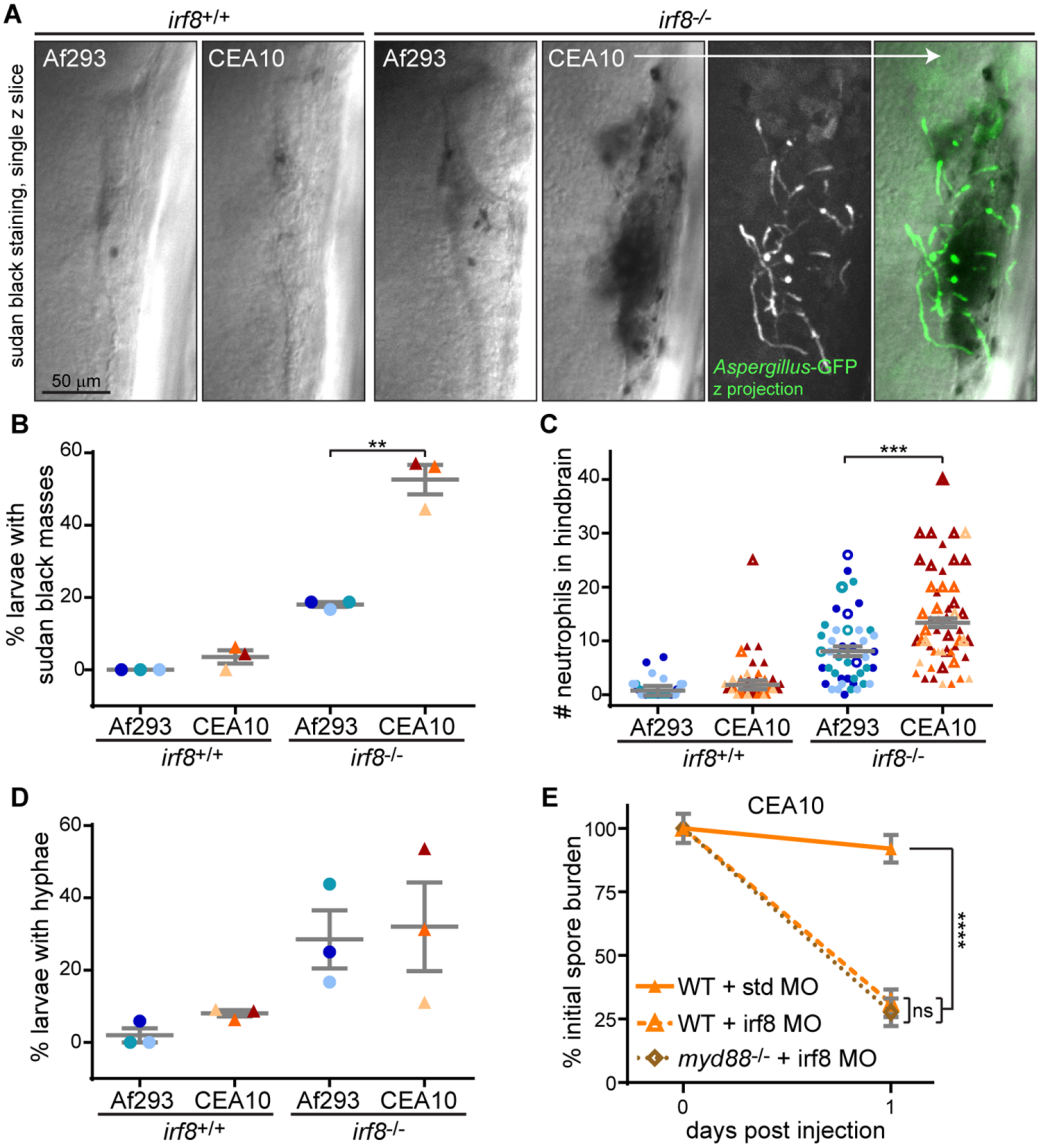
More neutrophils are recruited to a CEA10-derived strain than an Af293-derived strain in the absence of macrophages. **A-D**. Macrophage-deficient (*irf8*^-/-^) or control (*irf8*^+/+^) larvae were infected with YFP- or GFP-expressing *A*. *fumigatus* TBK1.1 (Af293) or TFYL49.1 (CEA10) strains, fixed 1 dpi, and stained for neutrophils (sudan black). Representative images are shown (A, scale bar represents 50 μm) and percent of larvae per experiment with sudan black masses (B), number of neutrophils at infection site (C), and percent of larvae per experiment with *A*. *fumigatus* hyphae (D) were quantified. N>9 larvae each condition each replicate. For percentage of larvae with sudan black masses or hyphae (B, D), each symbol represents one replicate, bars represent means ± SEM, P values calculated by t test. For neutrophil number quantification (C), each symbol represents one larvae. Larvae with sudan black masses where neutrophil numbers were difficult to count and represent underestimations are denoted with open symbols. Bars represent lsmeans ± SEM from 3 pooled replicates, P values calculated by ANOVA. All symbols are color-coded by replicate. **E**. Wild-type or *myd88*^-/-^ embryos were injected with control (std) or macrophage-depleting (irf8) morpholinos. Larvae were then infected with TFYL49.1 (CEA10) spores and CFUs were measured. Average injection CFUs = 38. CFU data represent lsmeans ± SEM of 3 pooled replicates, n = 24 larvae (3 replicates, 8 larvae each) per condition per day, P values calculated with ANOVA. See also S7 Fig.

To quantify neutrophil recruitment in the absence of macrophages, we performed sudan black staining 1 dpi in macrophage-deficient *irf8*^-/-^ or control larvae (Fig 7A–7C). Strikingly, in ~50% of macrophage-deficient larvae infected with a CEA10-derived strain, we observed extensive neutrophil recruitment that resulted in a mass of sudan black staining (Fig 7A and 7B). These sudan black masses were also sometimes observed in macrophage-deficient larvae infected with an Af293-derived strain, but significantly less so, and were rarely observed in control infected larvae. We quantified the number of neutrophils recruited to the infection site, finding significantly more recruitment in *irf8*^-/-^ larvae infected with an CEA10-derived strain compared to an Af293-derived strain (Fig 7C). These data indicate that in the absence of macrophages, high numbers of neutrophils are specifically recruited to CEA10 infections.

### Macrophages inhibit *A*. *fumigatus* germination

These results raise the question of what signals promote neutrophil recruitment to CEA10 in the absence of macrophages. Since neutrophils have been reported to respond specifically to hyphal forms of *A*. *fumigatus* [21], we hypothesized that macrophages inhibit spore germination. Indeed, the massive neutrophil recruitment we observed in *irf8*^-/-^ macrophage-deficient larvae was often accompanied by fungal germination and hyphal growth (Fig 7A). In fact, the percentage of larvae with hyphae present was higher in *irf8*^-/-^ macrophage-deficient hosts compared to wild-type hosts after infection with either an Af293-derived or a CEA10-derived strain (Fig 7D), suggesting that the presence of macrophages inhibits *A*. *fumigatus* germination. Similar results were seen in clodronate liposome-injected larvae (S7A Fig). Previous experiments also suggest this rate of germination may be an underestimate for CEA10 infection since this fungus is cleared so effectively in *irf8*^-/-^ macrophage-deficient conditions (Fig 6A). These data suggest that in the absence of macrophages, fungal germination induces signals that recruit neutrophils.

### Myd88 is not required for neutrophil-mediated fungal killing

Germination unmasks fungal cell wall components such as β-glucans that can be sensed by the host through TLR and CLR pathways [18]. Given our previous observation that the signaling molecule Myd88 is required for full clearance of a CEA10 strain (Fig 2F), we hypothesized that Myd88 is involved in neutrophil recruitment to the site of infection after spore germination. While Myd88 has been shown to be dispensable for neutrophil-intrinsic anti-fungal activity, it can be required in other cell types such as epithelial cells for neutrophil recruitment to *A*. *fumigatus* infections [34]. To determine if neutrophil-mediated killing of CEA10 in the absence of macrophages requires Myd88 signaling, we combined the *myd88*^-/-^ mutant line with macrophage-deficiency (*irf8* morpholino). We performed CFU counts at just 1 dpi since neutrophil-mediated clearance of CEA10 in the absence of macrophages occurs rapidly (Fig 6A). Consistent with previous results, at 1 dpi CEA10 is not cleared in wild-type hosts, while Irf8 deficiency alone led to significantly increased clearance (Figs 7E and S7B). However, we observed virtually identical clearance in macrophage-deficient hosts that also lacked Myd88 (Figs 7E and S7B). These data demonstrate that Myd88 is not required for neutrophil-mediated killing of germinated spores of a CEA10-derived strain, but instead that neutrophil activation signals are likely transduced through other pathways.

### A CEA10-derived strain germinates earlier than an Af293-derived strain *in vivo*

**Fig 8:**
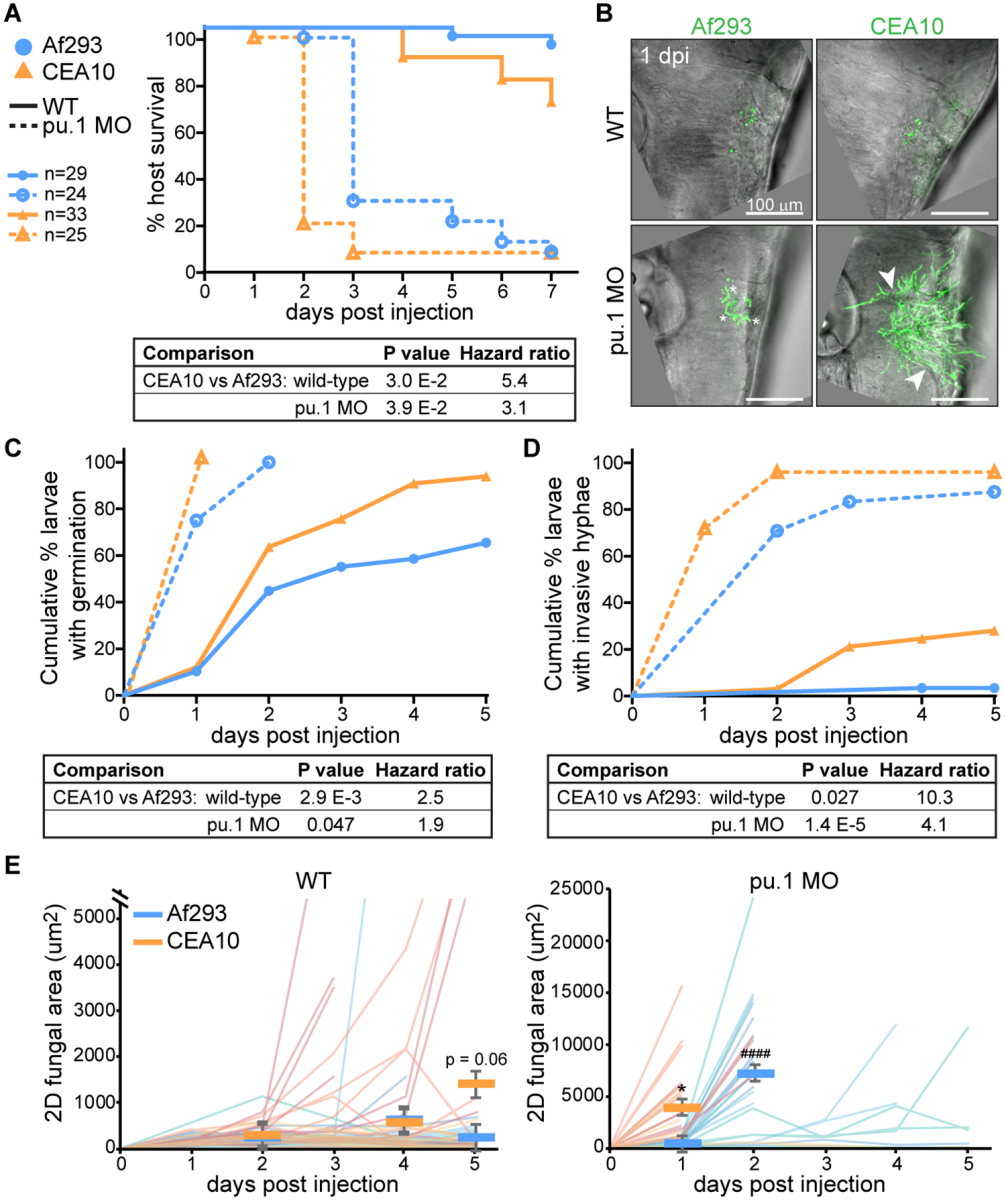
A CEA10-derived strain germinates faster than an Af293-derived strain *in vivo*. Wild-type or phagocyte-deficient (pu.1 morpholino) larvae were infected with YFP- or GFP-expressing *A*. *fumigatus* TBK1.1 (Af293) or TFYL49.1 (CEA10). All graphs represent data from 3 pooled replicates, Ns and labels noted in (A) are applicable for all data. **A**. Larval survival was monitored, P values calculated by Cox proportional hazard regression analysis. **B-E**. Larvae were imaged days 1–5 post injection and fungal growth was measured. Representative z-projection images 1 dpi are shown (B). Scale bar represents 100 μm. Asterisks indicate examples of germinated spores, arrowheads indicate examples of invasive hyphae. Cumulative percent of larvae with germinated spores (C) and with invasive hyphae (D) was calculated, P values were calculated by Cox proportional hazard regression analysis. 2D GFP^+^ fungal area was measured from maximum intensity projection images of individual larvae for 5 days of infection (or until larvae succumbed) (E). Each line represents one larvae followed for the entire course of infection, lines are color-coded by replicate, bars represent pooled lsmeans ± SEM, P values were calculated by ANOVA. See also S8 Fig.

Given our findings that neutrophils respond primarily to germinated *A*. *fumigatus* spores and that neutrophils are much better fungal killers than macrophages, we hypothesized that the increased killing of CEA10 compared to Af293 might be attributable to differences in germination and hyphal growth of these strains. CEA10 has been shown to germinate faster than Af293 in liquid culture *in vitro* [28] and more germinated spores of CEA10 compared to Af293 were found in the lungs of infected mice [27], but germination rates of these two strains have not been directly measured *in vivo*. To measure fungal growth *in vivo*, we infected both wild-type and phagocyte-deficient (*pu*.*1* morphant) larvae with YFP- or GFP-expressing spores of strains derived from either CEA10 or Af293. Phagocyte-deficient larvae succumb quickly to infection with either strain, but on average CEA10 infection results in death one day earlier than Af293 infection (Fig 8A), suggesting CEA10 grows faster *in vivo*. Similar results were obtained with several CEA10- and Af293-derived strains (S8 Fig). To directly monitor fungal development and growth over time, we imaged these larvae every day for five days of infection (or until larvae succumbed). After only 1 day of infection, we observed pervasive growth of CEA10 in phagocyte-deficient hosts (Fig 8B). While both Af293 and CEA10 spores had germinated after 1 day, CEA10 had already developed invasive hyphae throughout the hindbrain. We quantified how long it took individual larvae to exhibit both germinated spores (Fig 8C) and invasive hyphae (Fig 8D). These data confirmed that CEA10 both germinates significantly faster and grows into invasive hyphae significantly faster than Af293 in both wild-type and phagocyte-deficient hosts (Fig 8C and 8D). Quantification of fungal burden by measuring GFP^+^ area from these images also reflected greater growth of CEA10 and a ~1 day delay in Af293 growth (Fig 8E).

### Germination drives neutrophil-mediated fungal killing

**Fig 9:**
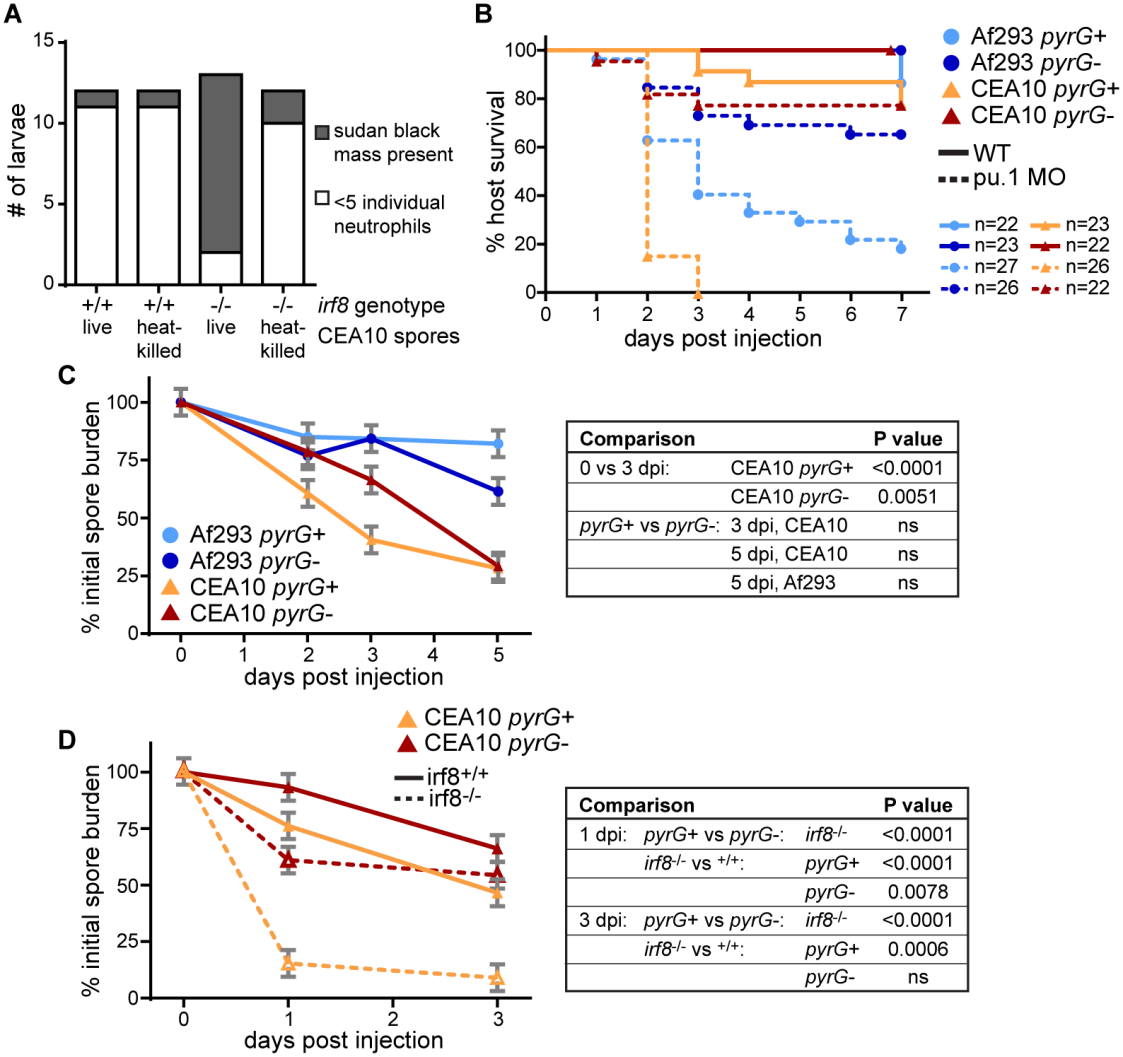
Neutrophil-mediated killing requires germination. **A**. Macrophage-deficient (*irf8*^-/-^) or control (*irf8*^+/+^) larvae were injected with live or heat-killed TFYL49.1 (CEA10), fixed 1 dpi, and stained for neutrophils (sudan black). Larvae were then scored for the presence or absence of a sudan black cluster or mass. This experiment was performed twice, data are shown from one representative replicate. **B**. Wild-type or phagocyte-deficient (pu.1 morphant) larvae were infected with TBK1.1 (Af293 *pyrG*+), Af293.1 (Af293 *pyrG*-), TFYL49.1 (CEA10 *pyrG*+), or CEA17 (CEA10 *pyrG*-) spores and survival was monitored. Data represent 2 pooled experiments. Average injection CFUs: Af293 *pyrG*+ = 52, Af293 *pyrG*- = 52, CEA10 *pyrG*+ = 38, CEA10 *pyrG*- = 48. **C**. Wild-type larvae were infected with TBK1.1 (Af293 *pyrG*+), Af293.1 (Af293 *pyrG*-), TFYL49.1 (CEA10 *pyrG*+), or CEA17 (CEA10 *pyrG*-) and CFUs were monitored. Average injection CFUs: Af293 *pyrG*+ = 45, Af293 *pyrG*- = 48, CEA10 *pyrG*+ = 42, CEA10 *pyrG*- = 49. **D**. Macrophage-deficient (*irf8*^-/-^) larvae were infected with CEA17 KU80Δ (CEA10 *pyrG*+), or CEA17 (CEA10 *pyrG*-) and CFUs were monitored. Average injection CFUs: CEA10 *pyrG*+ = 41, CEA10 *pyrG*- = 45. All CFU data represent lsmeans ± SEM from 3 pooled replicates composed of 24 larvae (8 larvae per replicate) per condition per day. P values were calculated by ANOVA. See also S9 Fig.

To investigate the role of germination in neutrophil recruitment, we injected heat-killed spores into macrophage-deficient *irf8*^-/-^ larvae and quantified the presence of sudan black masses in the hindbrain that we observed previously (Fig 7A and 7B). We found that heat-killed spores do not recruit neutrophils, suggesting that spore germination and fungal growth is required for neutrophil recruitment (Fig 9A).

However, immune cell killing activity against heat-killed spores cannot be measured, therefore to investigate the role of germination in fungal clearance, we utilized *A*. *fumigatus pyrG*-deficient strains, which are auxotrophic for uridine-uracil and are known to have impaired germination and attenuated virulence in mice [39]. To determine if these strains also have impaired germination in larval zebrafish we infected phagocyte-deficient larvae (*pu*.*1* morphant) and measured survival as well as germination. Indeed, germination of a CEA10-derived *pyrG*-deficient strain is abrogated in larval zebrafish and both CEA10- and Af293-derived *pyrG*-deficient strains cause minimal death in both wild-type and phagocyte-deficient larvae (Figs 9B and S9).

We infected wild-type larvae with Af293 and CEA10 *pyrG*- and *pyrG*+ reconstituted strains and performed CFU counts to determine the requirement for germination in fungal killing in the presence of both macrophages and neutrophils. Again, Af293 was not cleared significantly and the *pyrG* mutation did not have a significant effect on fungal burden in this strain background (Fig 9C). In a CEA10 infection, both a *pyrG*+ strain and *pyrG*- strain had significant levels of killing by 3 dpi and CFU counts from larvae infected with these strains were virtually identical 5 dpi (Fig 9C). These data suggest that in wild-type larvae germination is not required for fungal clearance, although we cannot rule out the possibility that *pyrG*-deficient spores do eventually germinate at later time points in infection.

**Fig 10:**
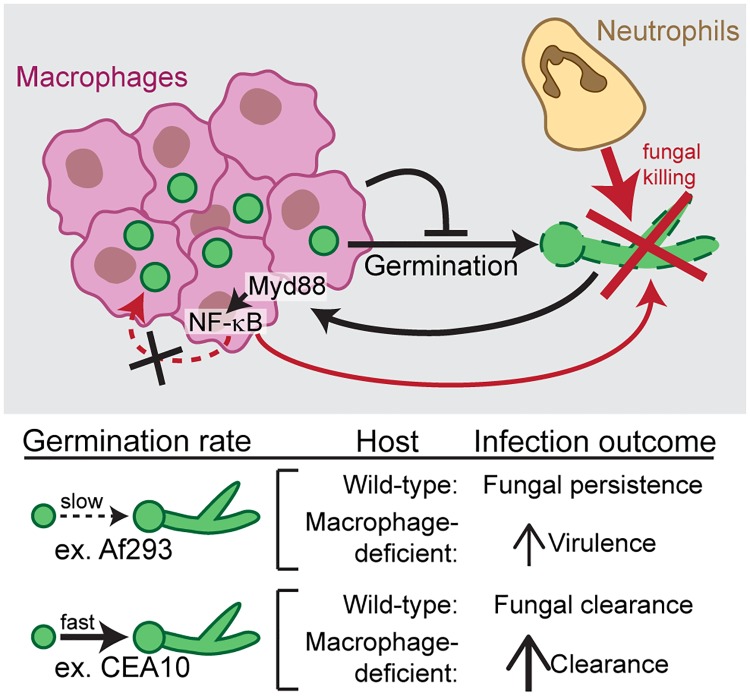
Model of neutrophil and macrophage responses to *Aspergillus fumigatus*. Upon infection, *A*. *fumigatus* spores are taken up by macrophages, which form dense clusters around the fungus and inhibit spore germination. Macrophages provide a protective niche for spore survival, as neutrophil-mediated killing of *A*. *fumigatus* requires germination. As a result, in infections with slow-germinating *A*. *fumigatus* strains, the fungus persists for days, while in infections with fast-germinating *A*. *fumigatus* strains, germination drives neutrophil recruitment, neutrophil-mediated killing, and faster fungal clearance. Macrophages have some anti-fungal activity, dependent on Myd88-NF-κB signaling, however NF-κB activation is not sufficient to kill slow-germinating strains.

To determine the requirement for germination in neutrophil-mediated killing, we determined the effect of *pyrG* deficiency in the specific context of CEA10 killing in *irf8*^-/-^ macrophage-deficient hosts (Fig 6A). In these larvae, neutrophils effectively clear ~85% of CEA10 *pyrG*+ fungal burden by 1 dpi (Fig 9D). However, a CEA10 *pyrG*- infection is cleared significantly less in this context. At 3 dpi, there is no difference in *pyrG*- fungal clearance between *irf8*^-/-^ macrophage-deficient larvae and wild-type larvae. These data indicate that *pyrG*-deficient CEA10 cannot be cleared by neutrophils, even in the absence of macrophages, suggesting that neutrophil-mediated clearance of CEA10 is dependent on germination. Together, these data demonstrate that germination drives neutrophil-mediated killing of *A*. *fumigatus*, and explain why a faster germinating strain is actually cleared more effectively by innate immune cells than a slower-growing strain (Fig 10).

## Discussion

Here we have used larval zebrafish to image *Aspergillus fumigatus* infection in live, intact hosts, over the course of a multi-day infection to visualize and quantify both immune cell behavior and fungal development and growth. Using a method to visually quantify spore killing, we find that faster-growing CEA10-derived strains of *A*. *fumigatus* are cleared significantly better than slower-growing Af293-derived strains. We report that clearance of CEA10-derived strains is primarily due to neutrophil function and is driven by fungal germination, whereas slower-growing Af293-derived strains do not recruit as many neutrophils and therefore can persist in the host for >5 days. Many fungal pathogens can survive within macrophages [40], but the role of macrophages *in vivo* in *A*. *fumigatus* pathogenesis has remained ambiguous, and we find that macrophages form tight clusters around injected spores. Macrophages inhibit spore germination, and without spore germination, neutrophil recruitment and neutrophil-mediated killing are diminished, thus macrophages act as a protective niche against neutrophil-mediated killing (Fig 10).

The phagocyte clusters that we observe are reminiscent of aspergillomas or “fungal granulomas” that are found in lung or intracranial lesions of human patients [41, 42]. These macrophage-driven phagocyte clusters have never been observed before in an animal model of *Aspergillus* infection, but phagocyte clustering is a common phenomenon in infections, with the mycobacteria granuloma as the classic example [43]. We do find some neutrophils infiltrating into these clusters and neutrophil aggregates have been reported in response to both to *Aspergillus* [12] and *Candida* [44]. Zebrafish larvae have been an ideal model in which to detect phagocyte clusters, and they have been found in response to not just mycobacteria [45], but also *Streptococcus iniaie* [46] and *Mucor circinelloides* [47].

In tuberculosis, granulomas can provide bacterial-protective functions [48] and macrophages can also act as protective niches for other pathogens [49]. *In vivo*, in larval zebrafish, macrophages do not have much spore killing ability, in contrast to what has been found *in vitro* [4, 5], but in line with some *in vivo* mouse experiments [6]. While in other mouse experiments macrophage depletion led to an increase in *A*. *fumigatus* fungal burden [7], we hypothesize that the protective niche function of macrophages is only relevant at lower doses of spores, not at the dose given in these experiments (~10^7^). Our data suggest that the protective niche function of macrophages arises simply by inhibition of fungal germination (Fig 10). While it is also possible that macrophages actively repel neutrophils, as we have observed this behavior in other inflammatory contexts [50], we cannot conclude this from the data presented here.

Neutrophils are highly efficient killers of *A*. *fumigatus*, and we find that this killing is largely dependent on germination. Whether neutrophils respond directly to signals exposed on hyphae or if epithelial cells and/or macrophages sense hyphae and recruit neutrophils in this context is not known. In mice, the neutrophil response to CEA10 is dependent on IL-1α production [27]. IL-1 receptor (IL-1R) signaling requires the Myd88 adaptor protein, but our experiments with *myd88*^-/-^ larvae demonstrate that Myd88 is not required for neutrophil recruitment and killing in this context. The Card9 adaptor can also transduce fungal signals to recruit neutrophils [34], and we hypothesize that this signaling is occurring in macrophage-deficient larvae to recruit neutrophils. Interestingly, in mice, neutrophil recruitment to Af293 and CEA10 infections is driven by different pathways and signals, in agreement with the differences we observe in the ability of these strains to recruit neutrophils [27].

In addition to inducing neutrophil recruitment, germination likely also makes *A*. *fumigatus* more susceptible to host killing mechanisms *in vivo*. In fact, we find that a slower-germinating Af293-derived strain is not effectively killed by the host even in a co-infection with a faster-germinating CEA10-derived strain. Hyphal growth and branching increases susceptibility to neutrophil-mediated killing *in vitro* [51], and it has long been thought that neutrophils are much more effective killers against hyphae than conidia [52]. One immune mechanism that neutrophils use against hyphae is neutrophil extracellular traps (NETs), which are observed in infected mouse lung tissue containing hyphae [20, 53]. In the absence of macrophages and the increased presence of hyphae we observed large masses of sudan black staining at the site of infection that are reminiscent of NETs. Sudan black stains neutrophil granules, and therefore these masses could be actual NETs, neutrophil degranulation, or simply massive neutrophil recruitment. It is worth noting that in imaging neutrophils expressing a cytosolic fluorescent protein recruited to the site of infection we could sometimes see a haziness in that channel suggesting neutrophil lysis, but could not prove the origin of this signal.

Differences in virulence between Af293 and CEA10 are also observed in murine models of *Aspergillus* infection. In a chemotherapeutic model of immunosuppression, infection with either of these strains caused similar rates of host death [26], a situation that may be similar to our dexamethasone-treated zebrafish larvae (Fig 2C). In a less immunosuppressed triamcinolone-treated mouse model, CEA10 is more virulent [26]. However, this increase in virulence is thought to be a result of increased resistance to hypoxia, a hallmark of this model [26], while we do not know the hypoxic state of our zebrafish immunosuppression models. Murine studies have correlated the increased growth and germination of CEA10 compared to Af293 with increased tissue damage [27], but have also found that increased growth *in vitro* across multiple environmental and clinical isolates correlates with increased host survival in the triamcinolone-treated mouse model [26], possibly consistent with our findings that increased growth leads to increased host response and clearance mechanisms.

Between these strains of *A*. *fumigatus*, we have identified two different virulence mechanisms, both of which are commonly observed in a variety of human pathogens. CEA10-derived strains can germinate quickly *in vivo*, and therefore under neutrophil-deficient conditions can cause disease and spread. On the other hand, Af293-derived strains do not germinate quickly but instead can persist largely undetected in the host. This latter mechanism is similar to one proposed for a different *Aspergillus* species, *A*. *terreus* [54], and for the fungal pathogen *Cryptococcus neoformans* [55]. These two different mechanisms highlight the contradiction underlying *Aspergillus* pathogenesis: while germination is required for virulence, it simultaneously activates the immune system and immune mechanisms that can kill the fungus. These ideas are relevant for the development and deployment of anti-*Aspergillus* treatments. Are there particular host scenarios in which it is better to simply inhibit germination and allow *Aspergillus* spores to persist? Or others where it is better to induce germination to activate host immune responses? Drugs that target macrophages to both maintain their ability to inhibit germination and increase neutrophil recruitment could provide the best possible scenario for future immunotherapies. Future investigation of the mechanisms behind each of these macrophage behaviors will be required to further understand the molecules and pathways involved.

## Methods

### Ethics statement

Animal care and use protocol M005405-A02 was approved by the University of Wisconsin-Madison College of Agricultural and Life Sciences (CALS) Animal Care and Use Committee. This protocol adheres to the federal Health Research Extension Act and the Public Health Service Policy on the Humane Care and Use of Laboratory Animals, overseen by the National Institutes of Health (NIH) Office of Laboratory Animal Welfare (OLAW).

### Zebrafish lines and maintenance

All zebrafish were maintained as described previously [56]. Prior to any experimental manipulation, larvae were anesthetized in E3 water containing 0.2 mg/ml Tricaine (ethyl 3-aminobenzoate, Sigma).

All zebrafish lines used in this study are listed in Table 1. Previously published lines were genotyped as described in referenced publications. To generate *lyz*:*BFP* larvae, *BFP* was amplified by PCR (F: 5’-cagtgatacaggtacctcgccaccatgagcgagctgattaagg-3’, R: 5’-ctgattatgatctagatcacttgtgccccagtttgctagg-3’) and inserted by In-Fusion HD Cloning (Clontech) into a Tol2-lyz vector [60, 61] cut with KpnI/XbaI. ~90 pg of Tol2 plasmid was micro-injected with 75 pg of transposase mRNA into embryos at the single cell stage. mRNA was *in vitro* transcribed (mMESSAGE mMACHINE SP6 kit, Ambion) from a pCS2-transposase vector [62]. Larvae were screened for BFP expression in neutrophils at 2–3 days post fertilization (dpf) and grown up to establish a stable line.

**Table 1:** Zebrafish lines used in this study.

Line name	Reference
*myd88*^-/-^	[57]
*irf8*^-/-^	[38]
Tg*(NF-κB RE*:*GFP)*	[33]
Tg*(lyz*:*BFP)*	This study
Tg*(mpeg1*:*H2B-mCherry)*	[56]
Tg*(mfap4*:*tomato-caax)*	[58]
Tg*(mpx*:*mCherry-2A-rac2D57N)*	[37]
Tg*(mpx*:*mCherry-2A-rac2WT)*	[37]
Tg(*mpx*:*mCherry)*	[59]

### Morpholino injections

Previously published and established splice-blocking morpholino oligonucleotides (MO, GeneTools) were re-suspended in water to a stock concentration of 1 mM. MOs were then diluted in water with 0.5X CutSmart Buffer (NEB) and 0.1% phenol red and 3 nl was micro-injected into single-cell stage embryos. Injected concentrations of morpholinos were 0.4 mM (*irf8* (ZFIN MO1: aatgtttcgcttactttgaaaatgg)[63]), 0.5 mM (*pu*.*1* (ZFIN MO1: gatatactgatactccattggtggt)[64]), and 0.33 mM (*myd88* (ZFIN MO2: gttaaacactgaccctgtggatcat)[65]). These injected amounts of *irf8* and *pu*.*1* MO are standard and efficacy of knockdown was previously confirmed [63, 64]. To confirm the splice-blocking effect of the *myd88* morpholino at this concentration, RNA was isolated from individual larvae with TRIzol reagent (Invitrogen), and cDNA was synthesized with SuperScript III RT and oligo-dT (Invitrogen). PCR was then performed with GoTaq (Promega) and primers for *myd88* (F: 5’-atggcatcaaagttaagtatagacc-3’, R: 5’-agggcagtgagagtgctttg-3’) and *ef1a* (F: 5’-tgccttcgtcccaatttcag-3’, R: 5’-taccctccttgcgctcaatc-3’)[66]. Shifts in band size were evaluated by agarose gel electrophoresis. For *irf8* and *myd88* MO injections, a standard control MO (GeneTools) was used at a matching concentration.

### Clodronate liposome injection

At 1.5 dpf, 2 nl of clodronate or PBS liposomes (Liposoma) with 0.1% phenol red was micro-injected intravenously into the caudal vein plexus of transgenic larvae expressing a fluorescent macrophage marker. Macrophage depletion was confirmed 24 hours later by loss of signal on a fluorescent zoomscope (EMS3/SyCoP3; Zeiss; Plan-NeoFluar Z objective) prior to hindbrain injection of *A*. *fumigatus*.

### *Aspergillus fumigatus* growth and spore microinjection

All *A*. *fumigatus* strains used in this study are listed in Table 2. TBK4.1 was generated by transforming TFYL44.1 with pJMP4, encoding the *A*. *fumigatus argB* gene [72]. TBK5.1 was generated from TBK4.1 by fusing mCherry to the 3’ end of *A*. *fumigatus gpdA* and inserting the *A*. *fumigatus pyrG* gene, as described previously for YFP expression in TBK1.1 [21]. Spores were grown at 37°C on solid glucose minimal media (GMM) and prepared for injection as described previously [21]. For *pyrG1* auxotrophs, 5.2 mM uridine and 5 mM uracil were added to GMM as supplements. Micro-injection of spore preparations into the hindbrain of 2 dpf larvae was performed as previously described [21]. We aimed for an infectious dose of ~30 spores per larvae. Actual spore injection doses were monitored by single larvae CFU platings on day 0 of infection as described below and are reported in figure legends for each experiment. Spores were heat-killed by incubation at 99°C for 30 min, with 300 rpm shaking. For survival and CFU experiments, individual larvae were maintained in 96-well plates. For imaging analyses, larvae were kept in 35 mm dishes or in 48-well plates.

**Table 2:** *Aspergillus fumigatus* strains used in this study.

Parental Background	Strain	Genetic background	Reference
Af293	Af293.1	*pyrG1*	[67]
	TJW55.2	*pyrG1*, *A*. *parasiticus pyrG*	[68]
	TFYL81.5	*pyrG1*, *argB1*, *ΔakuA*::*mluc*, *A*. *fumigatus pyrG*, *A*. *fumigatus argB*	[30]
	TBK1.1	*pyrG1*, *gpdA*::*YFP*::*A*. *fumigatus pyrG*	[21]
	TFYL44.1	*pyrG1*, *argB1*, *ΔakuA*	[30]
	TBK4.1	*pyrG1*, *argB1*, *ΔakuA*, *A*. *fumigatus argB*	This study
	TBK5.1	*pyrG1*, *argB1*, *ΔakuA*, *A*. *fumigatus argB*, *gpdA*::*mCherry*::*A*. *fumigatus pyrG*	This study
CEA10	CEA17	*pyrG1*	[69]
	CEA17 KU80Δ	*pyrG1*, *ΔakuB*::*A*. *fumigatus pyrG*	[70]
	TFYL49.1	*pyrG1*, *ΔakuB*::*pyrG; gpdA(p)*::*fmqB*::*eGFP*::*A*. *fumigatus pyrG*	[71]

### CFU counts

Single larvae were placed in 1.5 ml microcentrifuge tubes with 90 μl of 1x PBS containing 500 μg/ml Kanamycin and 500 μg/ml Gentamycin and homogenized in a mini bead beater at maximum speed for 10–20 seconds. The entire volume was plated on a 10 cm GMM plate, incubated for two days at 37°C, and CFUs were counted. For each CFU experiment, ≥8 larvae were individually plated for each time point and each condition. For co-infection CFU experiments, fluorescence of colonies was visualized on a zoomscope (EMS3/SyCoP3; Zeiss; Plan-NeoFluar Z objective) to specifically count colonies of each strain. All CFU data were normalized to the average initial injection dose for each replicate and condition.

### Live-dead spore labeling

Spore cell walls were labeled with AlexaFluor molecules as previously described [10]. Briefly, biotin-XX, SSE (Life Technologies) was conjugated to spores in 0.05 M NaHCO_3_. Spores were washed in Tris-HCl pH 8 to deactivate free-floating biotin, then washed in PBS, and incubated with streptavidin-AF594 or -AF633 (Life Technologies). Labeling was confirmed by fluorescence imaging and spores were resuspended in PBS and injected as described above.

### Live imaging

To prevent pigment formation, 0.2 mM N-phenylthiourea (PTU, Sigma-Aldrich) was added to E3 water 1 dpf. Larvae carrying fluorescent transgenes were pre-screened on a zoomscope (EMS3/SyCoP3; Zeiss; Plan-NeoFluar Z objective) prior to the experiment. For daily imaging, larvae were maintained in single wells of a 48-well plate. Each day, larvae were removed, one at a time, anesthetized in E3 with Tricaine, and placed into zWEDGI chambers [73] to correctly orient their hindbrains towards the bottom of the dish. Images were acquired on a spinning disk confocal microscope (CSU-X; Yokogawa) with a confocal scanhead on a Zeiss Observer Z.1 inverted microscope, Plan-Apochromat NA 0.8/20x objective, and a Photometrics Evolve EMCCD camera. Images were acquired with ZEN software (Zeiss). After imaging was complete, larvae were washed in E3 with PTU and placed back into the same plate well. To image AlexaFluor-labeled spores, larvae were also imaged in zWEDGI chambers [73] on the same spinning disk confocal microscope with an EC Plan-Neofluar NA 0.75/40x objective. Clodronate-injected larvae were also imaged with the spinning disk confocal with a EC Plan-Neofluar NA 0.3/10x objective. To image GFP expression in the *NF-κB RE*:*GFP* line, 2 days post injection (dpi) larvae were mounted in a glass-bottom dish with 1% low-melting point agarose. Images were acquired with a laser-scanning confocal microscope (FluoView FV1000; Olympus) with an NA 0.75/20x objective and FV10-ASW software (Olympus). To image *mfap4*:*tomato-caax* larvae, larvae at 3 dpi (for macrophage cluster imaging) or 1 dpi (for co-infection imaging) were mounted in agarose and imaged on the Olympus laser-scanning confocal microscope as described above.

### Image analysis/processing

In all displayed images, image histogram levels were adjusted in Fiji. For any experiments where fluorescence intensity of images was quantified, no alteration was made to images prior to analysis. To quantify killing of AlexaFluor-labeled spores (Fig 1B and 1C), alive and killed spores were manually counted in Fiji. Displayed images of Alexa-Fluor labeled spores were also processed in Fiji with bilinear interpolation to increase the pixel density two-fold. To quantify GFP signal from the *NF-κB RE*:*GFP* zebrafish line (Fig 2A and 2B), a single slice from each z-stack representing the center of the hindbrain was isolated. In Fiji, the hindbrain region was manually identified in the corresponding brightfield image and the mean grey value as well as the integrated density (area × mean grey value) in that region in the GFP channel was measured. Displayed images of GFP signal were also processed in Fiji with bilinear interpolation to increase the pixel density two-fold. Signal intensity is displayed with the 16 colors look up table. To quantify phagocyte recruitment and fungal growth (Figs 4, 7 and 8), z-stacks and/or maximum intensity projections were analyzed in Fiji. Cell numbers, phagocyte cluster area, and presence of germination and/or invasive hyphae were scored manually. 2D fungal area was calculated by thresholding. Images of phagocyte recruitment (Fig 4C) were processed in Fiji with bilinear interpolation to increase the pixel density two-fold followed by a gaussian blur (radius = 0.75). Images in S1 Movie were processed in Fiji with bilinear interpolation to increase the pixel density two-fold followed by a gaussian blur (radius = 1). The displayed image of tomato+ clustered cells (Fig 4E) was processed in Fiji with bilinear interpolation to increase the pixel density four-fold followed by a gaussian blur (radius = 2). For co-infection imaging quantification (Fig 3), pair-wise distances between spores in each larvae were measured manually in Fiji. Displayed images of co-infection were processed in Fiji with bilinear interpolation to increase the pixel density two-fold followed by a gaussian blur (radius = 1). Whole larvae images of macrophages and neutrophils after clodronate injection (S6A Fig) were manually assembled. Images of *A*. *fumigatus* infected clodronate-depleted or control larvae (S7A Fig) were processed in Fiji with bilinear interpolation to increase the pixel density two-fold followed by a gaussian blur (radius = 0.75).

### Drug treatments

Dexamethasone (Sigma) was resuspended to a stock concentration of 10 mM in 100% ethanol. Larvae were treated with 10 μM dexamethasone, or equivalent ethanol control (0.1%) immediately after infection and the drug was left on larvae for the entirety of the experiment. Withaferin A was resuspended in DMSO to a stock concentration of 10 mM. 2 hpi, E3 was changed to E3 with 30 μM withaferin A (Santa Cruz Biotechnology), or DMSO (0.3%). 24 hpi, drug was removed and larvae were placed back in E3 alone.

### Sudan black staining

Sudan black staining was performed as described in protocol dx.doi.org/10.17504/protocols.io.rced2te. For visualization of sudan black staining (and YFP/GFP of injected fungus), larvae were placed into zWEDGI wells [73] to correctly orient their hindbrains and imaged on a spinning disk confocal microscope (CSU-X; Yokogawa) with a confocal scanhead on a Zeiss Observer Z.1 inverted microscope, Plan-Apochromat NA 0.8/20x objective, a Photometrics Evolve EMCCD camera, and ZEN software (Zeiss).

### Calcofluor white staining

Larvae were anesthetized and placed on a glass slide. As much liquid as possible was removed, 5 μl of 1 g/L calcofluor white was pipetted on top of the larvae, and a glass coverslip was placed on top. Smashed larvae were imaged for the presence of calcofluor-white stained hyphae within 10 minutes on a Zeiss Observer Z.1 inverted microscope with a EC Plan-Neofluar NA 0.30/10x objective and epifluorescence images were taken with a Photometrics Coolsnap ES^2^ camera and ZEN software (Zeiss).

### Statistical analyses

For all statistical analyses, three independent experiments were performed. Experimental Ns are noted in figures and/or figure legends. For larval survival data and analysis of cumulative germination and invasive hyphal occurrence in larvae, replicates were pooled and analysed by Cox proportional hazard regression analysis, with experimental replicate included as a group variable. In addition to pair-wise P values, this analysis also calculates hazard ratios, which are sometimes displayed. The hazard ratio can be interpreted as the relative death rate and represents the relative instantaneous risk of death throughout the experiment between two conditions. For quantification of imaging experiments (spore killing, intensity, cell counts, cluster area, or fungal area) and for CFU quantification, pooled data from three replicates was compared between experimental conditions using analysis of variance. Results are summarized in terms of least-squared adjusted means (lsmeans) and standard error (SEM). In some cases, graphs show both calculated lsmeans ± SEM and individual data points, color-coded by replicate. For data in Fig 7B and 7D, representing percent of larvae per experiment over three experiments, P values were calculated by t test and regular means ± SEM are displayed. Statistical analyses and graphical representations were done in R version 3.4, GraphPad Prism version 6, and/or Microsoft Excel 2016.

## Supporting information

S1 Fig Multiple CEA10-derived strains are cleared faster than multiple Af293-derived strains *in vivo*. Wild-type larvae were infected with Af293-derived strains (Af293, TBK1.1, TBK5.1) or CEA10-derived strains (CEA10, CEA17 KU80Δ, TFYL49.1) and fungal burden was monitored by CFUs. Average injection CFUs: Af293 = 58, TBK1.1 = 61, TBK5.1 = 61, CEA10 = 75, CEA17 KU80Δ = 63, TFYL49.1 = 46. CFUs from 24 larvae (3 replicates, 8 larvae each) per strain per day were measured. Data represent lsmeans ± SEM from three pooled experiments. P values comparing CFUs at 5 dpi calculated by ANOVA.
(TIF)

S2 Fig Inflammatory activation is only partially responsible for differences in spore killing. **A**. Larvae were treated with dexamethasone (DEX) or ethanol vehicle control directly after injection of PBS and survival was monitored. **B**. Larvae were injected with *A*. *fumigatus* Af293-derived (TJW55.2 or TBK1.1) or CEA10-derived (CEA17 KU80Δ or TFYL49.1) spores, treated with 30μM withaferin A or DMSO vehicle control from 2 hpi until 1 dpi, and survival was monitored. Average injection CFUs: Af293 = 47, CEA10 = 64. Data represent 3 pooled replicates, P values calculated by Cox proportional hazard regression analysis. **C**. RNA was isolated from 2 dpf larvae injected with *myd88* morpholino and RT-PCR was performed to monitor splice-blocking. Each lane is sample from a single larvae; *ef1a* is included as a loading control. **D**. Morpholino-injected (*myd88* or standard control) NF-κB RE:EGFP larvae were infected with non-fluorescent CEA17 KU80Δ (CEA10) spores and imaged 2 dpi. Quantification of signal from two replicates is shown. Each symbol represents one larvae, color-coded by replicate. Standard control n = 18; *myd88* n = 18.
(TIF)

S3 Fig Af923- and CEA10-derived spores co-localize when co-injected. Macrophage-membrane labeled larvae (*mfap4*:*tomato-CAAX*) were co-infected with YFP-expressing TBK1.1 (Af293) and AlexaFluor633-labeled CEA17 KU80Δ (CEA10) and imaged 1 dpi. A single z-slice image containing the entire hindbrain region of a representative larvae is shown. Scale bar represents 20 μm. Boxes indicate regions shown at higher magnification in Fig 3C.
(TIF)

S4 Fig Imaging of phagocyte clusters. **A**. Individual channels of images from Fig 4C are shown. Representative z projection images of dual macrophage-nuclear (*mpeg1*:*mcherry-H2B*) and neutrophil (*lyz*:*BFP*) labeled larvae infected with YFP- or GFP-expressing *A*. *fumigatus* TBK1.1 (Af293) or TFYL49.1 (CEA10) strains and imaged days 1 and 2 post injection. Scale bar represents 20 μm, inset scale bar represents 5 μm. Examples of spore germination inside the cluster are marked in insets with arrowheads. **B**. Nuclear macrophage labeled larvae (*mpeg1*:*mcherry-H2B*) were infected with GFP-expressing TFYL49.1 (CEA10). Z-projection (mcherry, GFP) or single slice (BF) images of the same larvae on days 2, 4, and 5 dpi are shown. Extrusion at 4 dpi is marked with an arrow. Scale bar represents 50 μm.
(TIF)

S5 Fig Initial spore dose does not affect clearance of a CEA10-derived strain in neutrophil-defective larvae. Neutrophil-defective (*mpx*:*rac2D57N*) or control (*mpx*:*rac2WT*) larvae were infected with two different doses of TFYL49.1 (CEA10) and CFUs were monitored. Average injection CFUs: “½ dose” = 19, “full dose” = 56. Data are from 24 larvae (3 replicates, 8 larvae each) per condition per day, lsmeans ± SEM from pooled replicates are shown, P values calculated by ANOVA.
(TIF)

S6 Fig Clodronate liposomes specifically deplete macrophages. **A**. Dual macrophage (*mpeg1*:*GFP*) and neutrophil (*mpx*:*mCherry*) labeled larvae were injected with clodronate liposomes, or left uninjected, and imaged 24 hours later. Z-projection (GFP, mcherry) or single slice (BF) images are shown. Scale bar represents 250 μm. **B**. PBS was injected into the hindbrains of clodronate liposome-injected or control (PBS liposomes and/or uninjected i.v.) larvae and survival was monitored. Data shown are from 3 pooled replicates.
(TIF)

S7 Fig Macrophage depletion leads to more germination and clearance of a CEA10-derived strain. **A**. Macrophage-depleted (clodronate liposomes) or control (uninjected i.v.) larvae were infected with GFP-expressing TFYL49.1 (CEA10) and imaged 1 dpi. Z-projection (mcherry, GFP) or single slice (BF) images shown are representative of 9/9 larvae from each condition from one experiment. Scale bar represents 100 μm or 25 μm (inset). **B**. Wild-type or *myd88*^-/-^ embryos were injected with control (std) or macrophage-depleting (irf8) morpholinos. Larvae were then infected with TFYL49.1 (CEA10) spores and CFUs were measured. Data are from one experiment, each symbol represents one larva. These data are also included in the pooled data from three replicates shown in Fig 7E.
(TIF)

S8 Fig Multiple CEA10-derived strains are more virulent than multiple Af293-derived strains in phagocyte-deficient larvae. Phagocyte-deficient (pu.1 morpholino) larvae were infected with Af293-derived strains (Af293, TBK1.1, TBK5.1) or CEA10-derived strains (CEA10, CEA17 KU80Δ, TFYL49.1), or injected with PBS, and survival was monitored. Average injection CFUs: Af293 = 60, TBK1.1 = 69, TBK5.1 = 54, CEA10 = 55, CEA17 KU80Δ = 67, TFYL49.1 = 54. Data represent 3 pooled replicates.
(TIF)

S9 Fig *pyrG*- strains are deficient in germination in larval zebrafish. Phagocyte-deficient larvae (pu.1 morphant) were infected with non-fluorescent spores of CEA17 KU80Δ (CEA10 *pyrG*+) or CEA17 (CEA10 *pyrG*-). 1 dpi *A*. *fumigatus* growth was visualized in flattened larvae with calcofluor white (CFW) staining and representative widefield images are shown. e = eye, ot = otic vesicle. Number of larvae with hyphal growth in 2 replicates was quantified.
(TIF)

S1 Movie Phagocyte clusters form around injected *Aspergillus*. Dual macrophage-nuclear (*mpeg1*:*mcherry-H2B*, magenta) and neutrophil (*lyz*:*BFP*, yellow) labeled larvae were infected with GFP-expressing TFYL49.1 (CEA10, green). Focus-through of a z stack of an infected larvae hindbrain is shown from imaging 2 dpi.
(MP4)
