# Supplementary figures and images for: Macrophages inhibit Aspergillus fumigatus germination and neutrophil-mediated fungal killing
Source: PLoS Pathog. 2018 Aug 2;14(8):e1007229. doi: 10.1371/journal.ppat.1007229 (PMC6091969; doi:10.1371/journal.ppat.1007229)

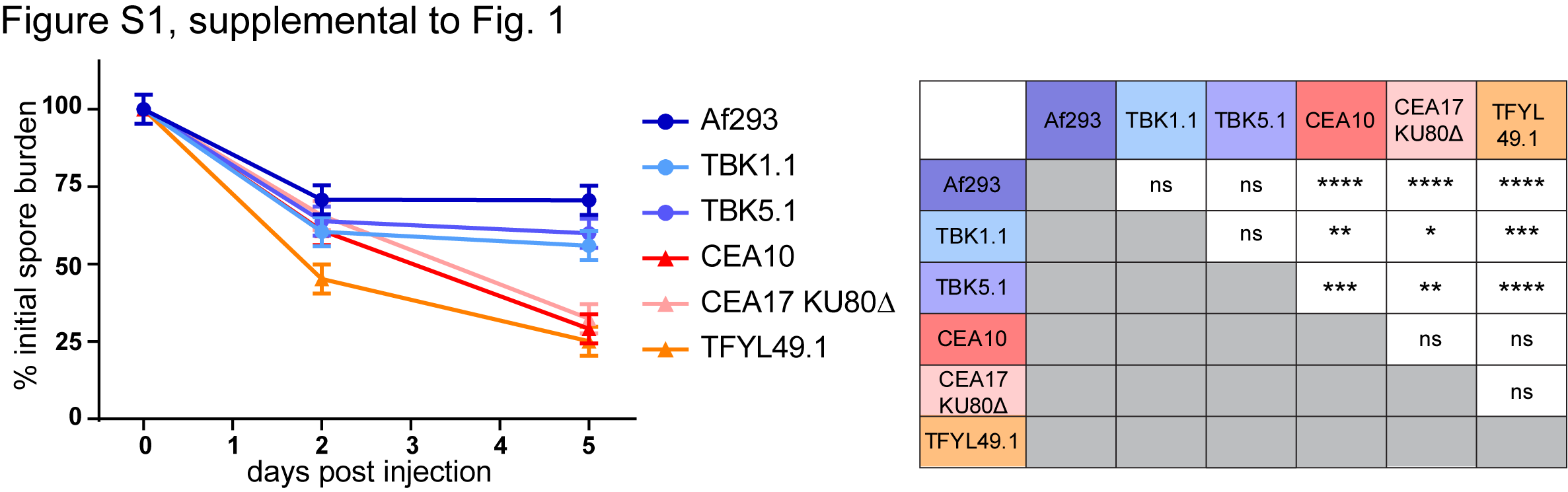

Supplement: S1 Fig — Wild-type larvae were infected with Af293-derived strains (Af293, TBK1.1, TBK5.1) or CEA10-derived strains (CEA10, CEA17 KU80Δ, TFYL49.1) and fungal burden was monitored by CFUs. Average injection CFUs: Af293 = 58, TBK1.1 = 61, TBK5.1 = 61, CEA10 = 75, CEA17 KU80Δ = 63, TFYL49.1 = 46. CFUs from 24 larvae (3 replicates, 8 larvae each) per strain per day were measured. Data represent lsmeans ± SEM from three pooled experiments. P values comparing CFUs at 5 dpi calculated by ANOVA. (TIF) [file ppat.1007229.s001.tif]

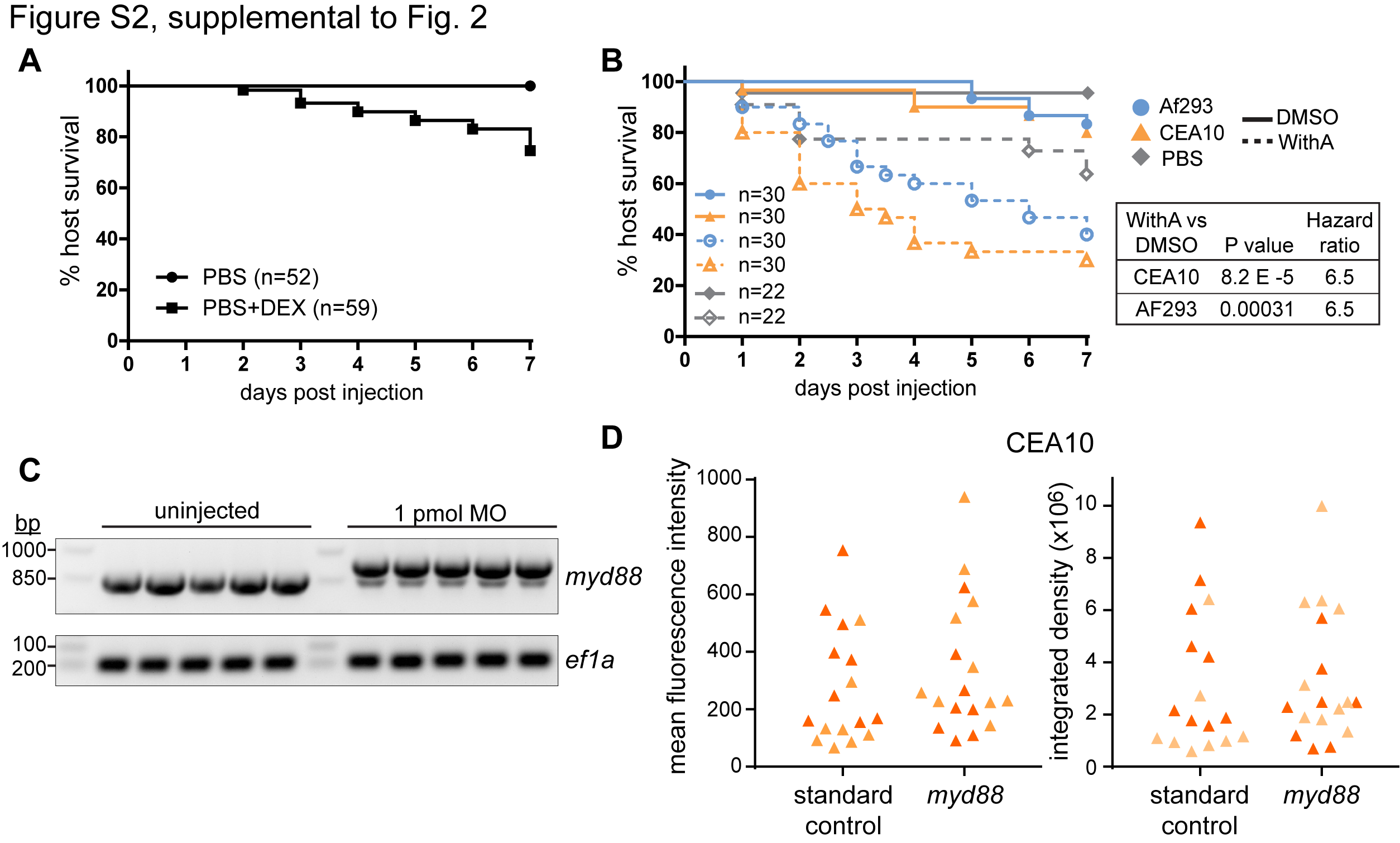

Supplement: S2 Fig — A. Larvae were treated with dexamethasone (DEX) or ethanol vehicle control directly after injection of PBS and survival was monitored. B. Larvae were injected with A. fumigatus Af293-derived (TJW55.2 or TBK1.1) or CEA10-derived (CEA17 KU80Δ or TFYL49.1) spores, treated with 30μM withaferin A or DMSO vehicle control from 2 hpi until 1 dpi, and survival was monitored. Average injection CFUs: Af293 = 47, CEA10 = 64. Data represent 3 pooled replicates, P values calculated by Cox proportional hazard regression analysis. C. RNA was isolated from 2 dpf larvae injected with myd88 morpholino and RT-PCR was performed to monitor splice-blocking. Each lane is sample from a single larvae; ef1a is included as a loading control. D. Morpholino-injected (myd88 or standard control) NF-κB RE:EGFP larvae were infected with non-fluorescent CEA17 KU80Δ (CEA10) spores and imaged 2 dpi. Quantification of signal from two replicates is shown. Each symbol represents one larvae, color-coded by replicate. Standard control n = 18; myd88 n = 18. (TIF) [file ppat.1007229.s002.tif]

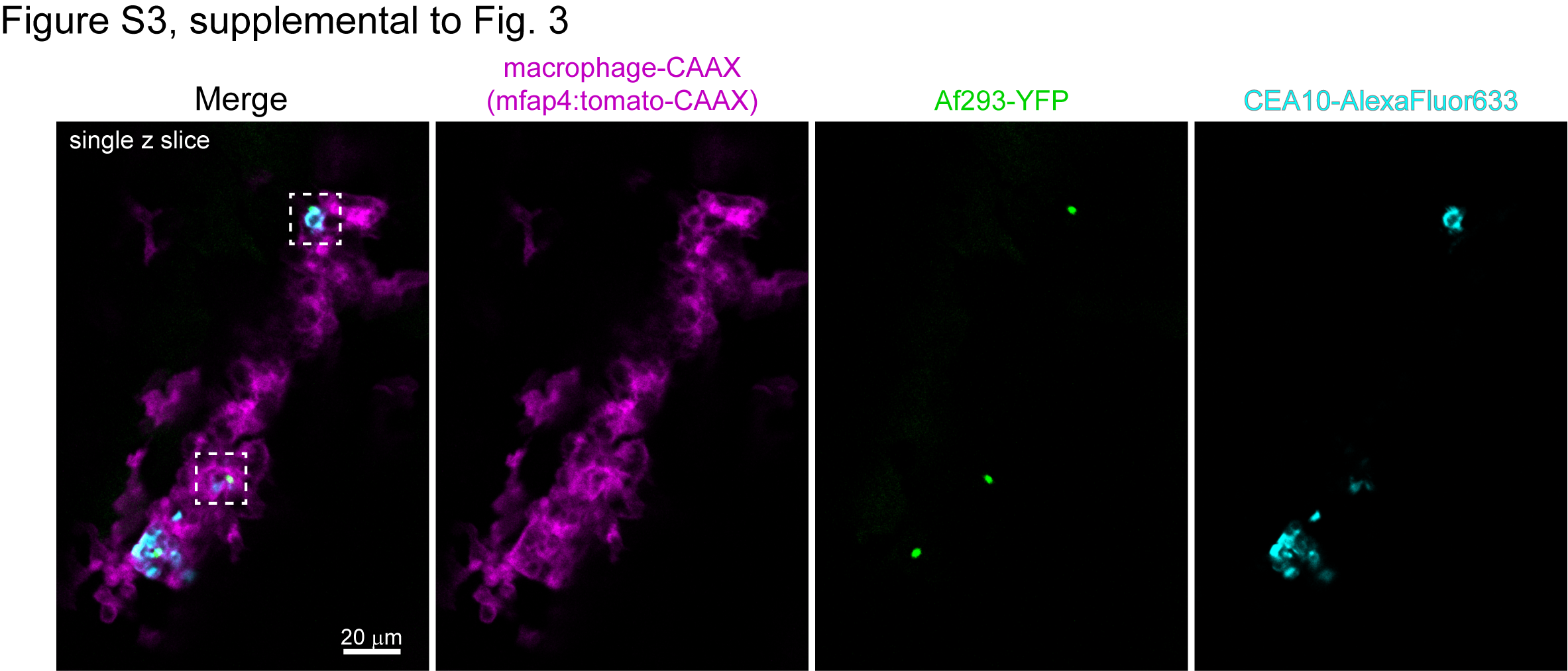

Supplement: S3 Fig — Macrophage-membrane labeled larvae (mfap4:tomato-CAAX) were co-infected with YFP-expressing TBK1.1 (Af293) and AlexaFluor633-labeled CEA17 KU80Δ (CEA10) and imaged 1 dpi. A single z-slice image containing the entire hindbrain region of a representative larvae is shown. Scale bar represents 20 μm. Boxes indicate regions shown at higher magnification in Fig 3C. (TIF) [file ppat.1007229.s003.tif]

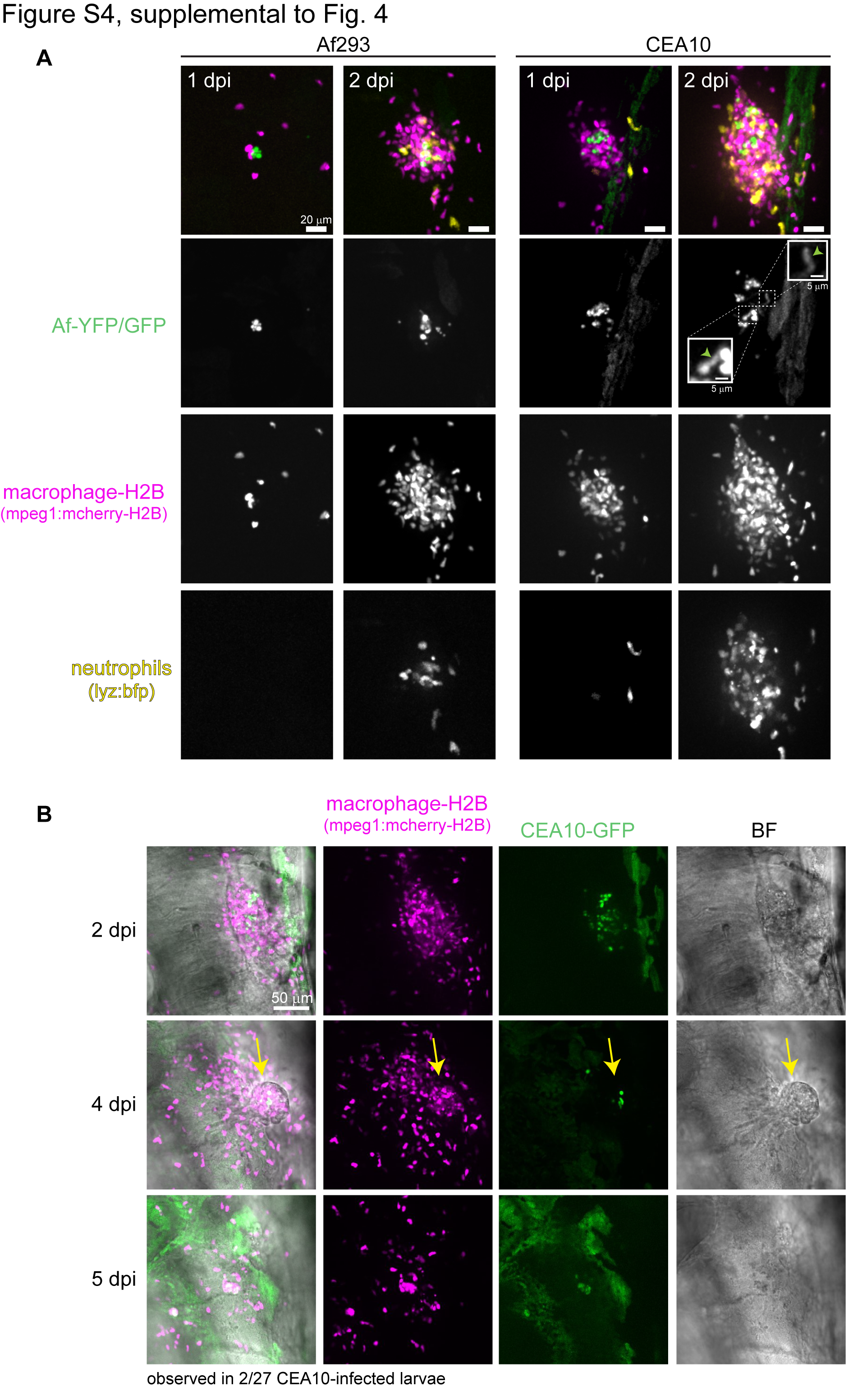

Supplement: S4 Fig — A. Individual channels of images from Fig 4C are shown. Representative z projection images of dual macrophage-nuclear (mpeg1:mcherry-H2B) and neutrophil (lyz:BFP) labeled larvae infected with YFP- or GFP-expressing A. fumigatus TBK1.1 (Af293) or TFYL49.1 (CEA10) strains and imaged days 1 and 2 post injection. Scale bar represents 20 μm, inset scale bar represents 5 μm. Examples of spore germination inside the cluster are marked in insets with arrowheads. B. Nuclear macrophage labeled larvae (mpeg1:mcherry-H2B) were infected with GFP-expressing TFYL49.1 (CEA10). Z-projection (mcherry, GFP) or single slice (BF) images of the same larvae on days 2, 4, and 5 dpi are shown. Extrusion at 4 dpi is marked with an arrow. Scale bar represents 50 μm. (TIF) [file ppat.1007229.s004.tif]

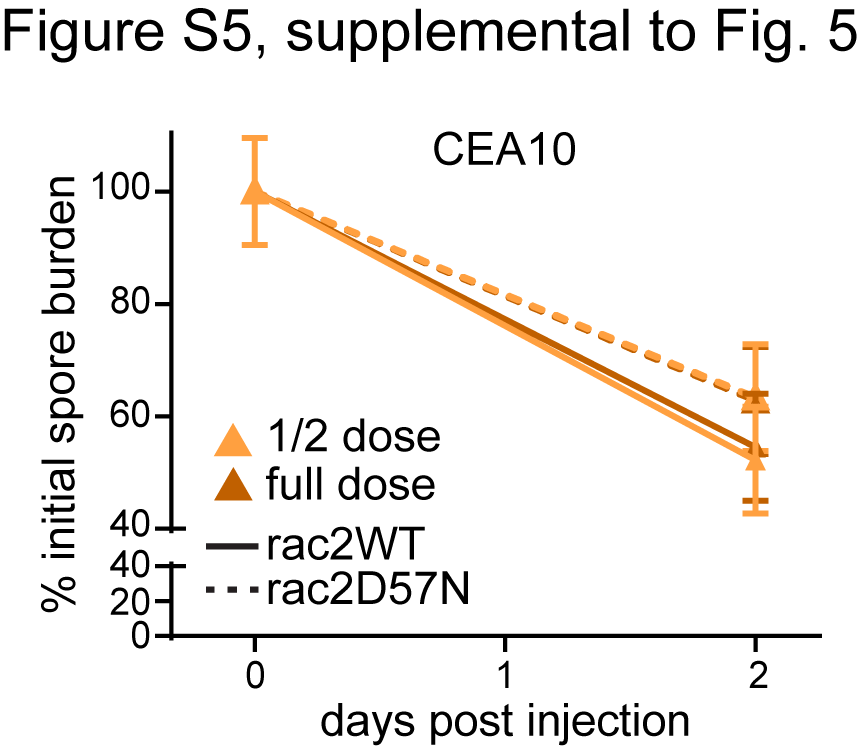

Supplement: S5 Fig — Neutrophil-defective (mpx:rac2D57N) or control (mpx:rac2WT) larvae were infected with two different doses of TFYL49.1 (CEA10) and CFUs were monitored. Average injection CFUs: “½ dose” = 19, “full dose” = 56. Data are from 24 larvae (3 replicates, 8 larvae each) per condition per day, lsmeans ± SEM from pooled replicates are shown, P values calculated by ANOVA. (TIF) [file ppat.1007229.s005.tif]

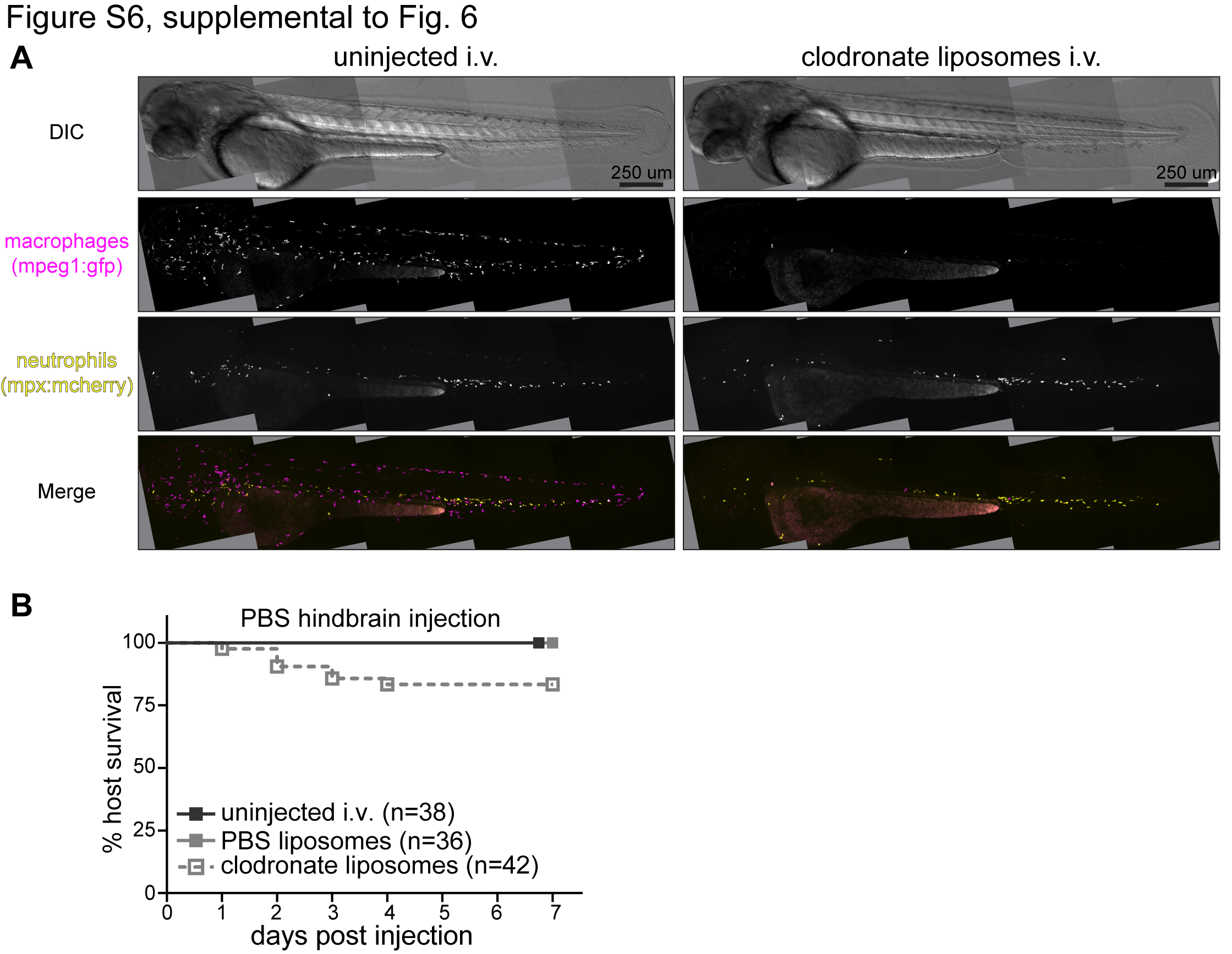

Supplement: S6 Fig — A. Dual macrophage (mpeg1:GFP) and neutrophil (mpx:mCherry) labeled larvae were injected with clodronate liposomes, or left uninjected, and imaged 24 hours later. Z-projection (GFP, mcherry) or single slice (BF) images are shown. Scale bar represents 250 μm. B. PBS was injected into the hindbrains of clodronate liposome-injected or control (PBS liposomes and/or uninjected i.v.) larvae and survival was monitored. Data shown are from 3 pooled replicates. (TIF) [file ppat.1007229.s006.tif]

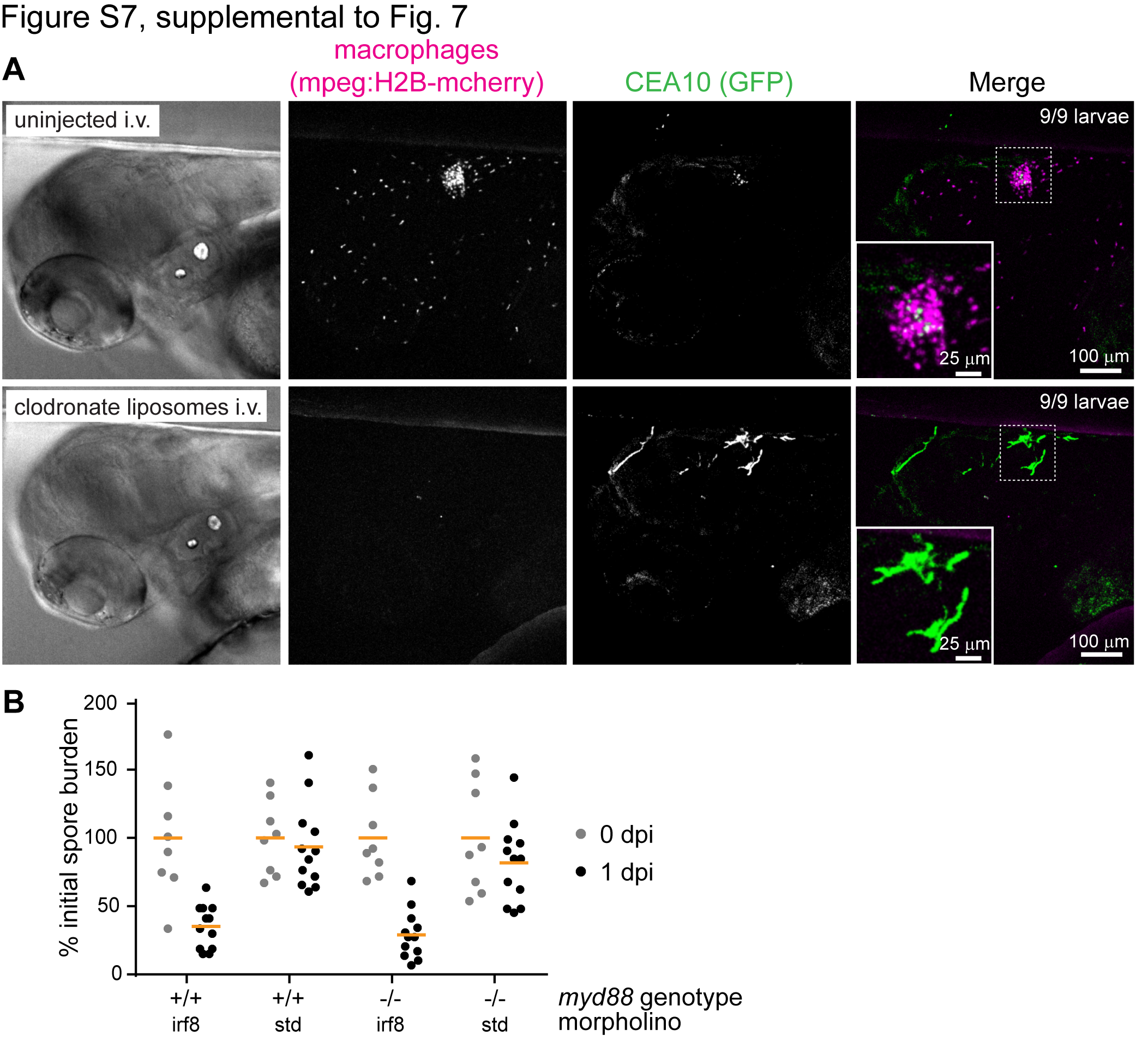

Supplement: S7 Fig — A. Macrophage-depleted (clodronate liposomes) or control (uninjected i.v.) larvae were infected with GFP-expressing TFYL49.1 (CEA10) and imaged 1 dpi. Z-projection (mcherry, GFP) or single slice (BF) images shown are representative of 9/9 larvae from each condition from one experiment. Scale bar represents 100 μm or 25 μm (inset). B. Wild-type or myd88-/- embryos were injected with control (std) or macrophage-depleting (irf8) morpholinos. Larvae were then infected with TFYL49.1 (CEA10) spores and CFUs were measured. Data are from one experiment, each symbol represents one larva. These data are also included in the pooled data from three replicates shown in Fig 7E. (TIF) [file ppat.1007229.s007.tif]

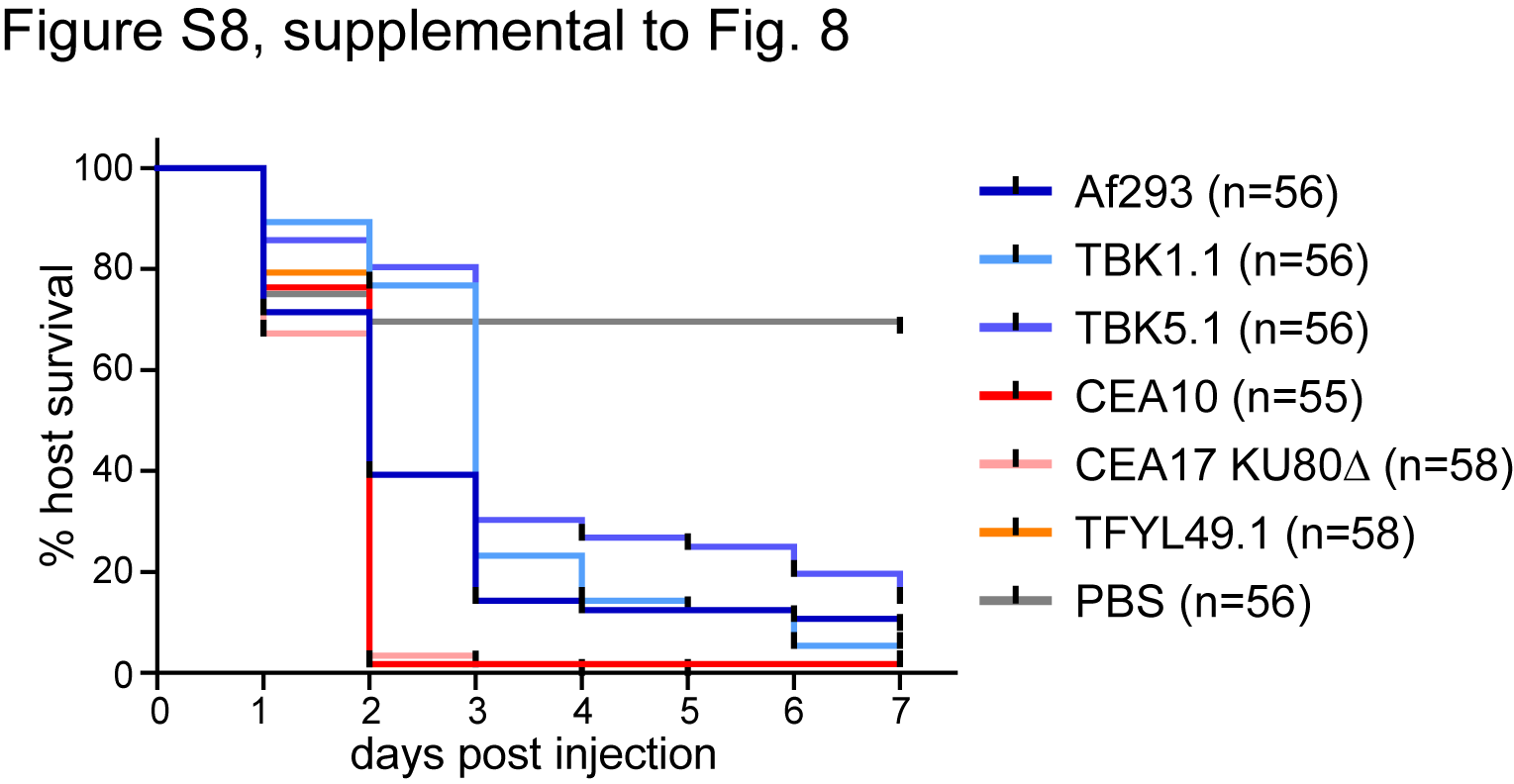

Supplement: S8 Fig — Phagocyte-deficient (pu.1 morpholino) larvae were infected with Af293-derived strains (Af293, TBK1.1, TBK5.1) or CEA10-derived strains (CEA10, CEA17 KU80Δ, TFYL49.1), or injected with PBS, and survival was monitored. Average injection CFUs: Af293 = 60, TBK1.1 = 69, TBK5.1 = 54, CEA10 = 55, CEA17 KU80Δ = 67, TFYL49.1 = 54. Data represent 3 pooled replicates. (TIF) [file ppat.1007229.s008.tif]

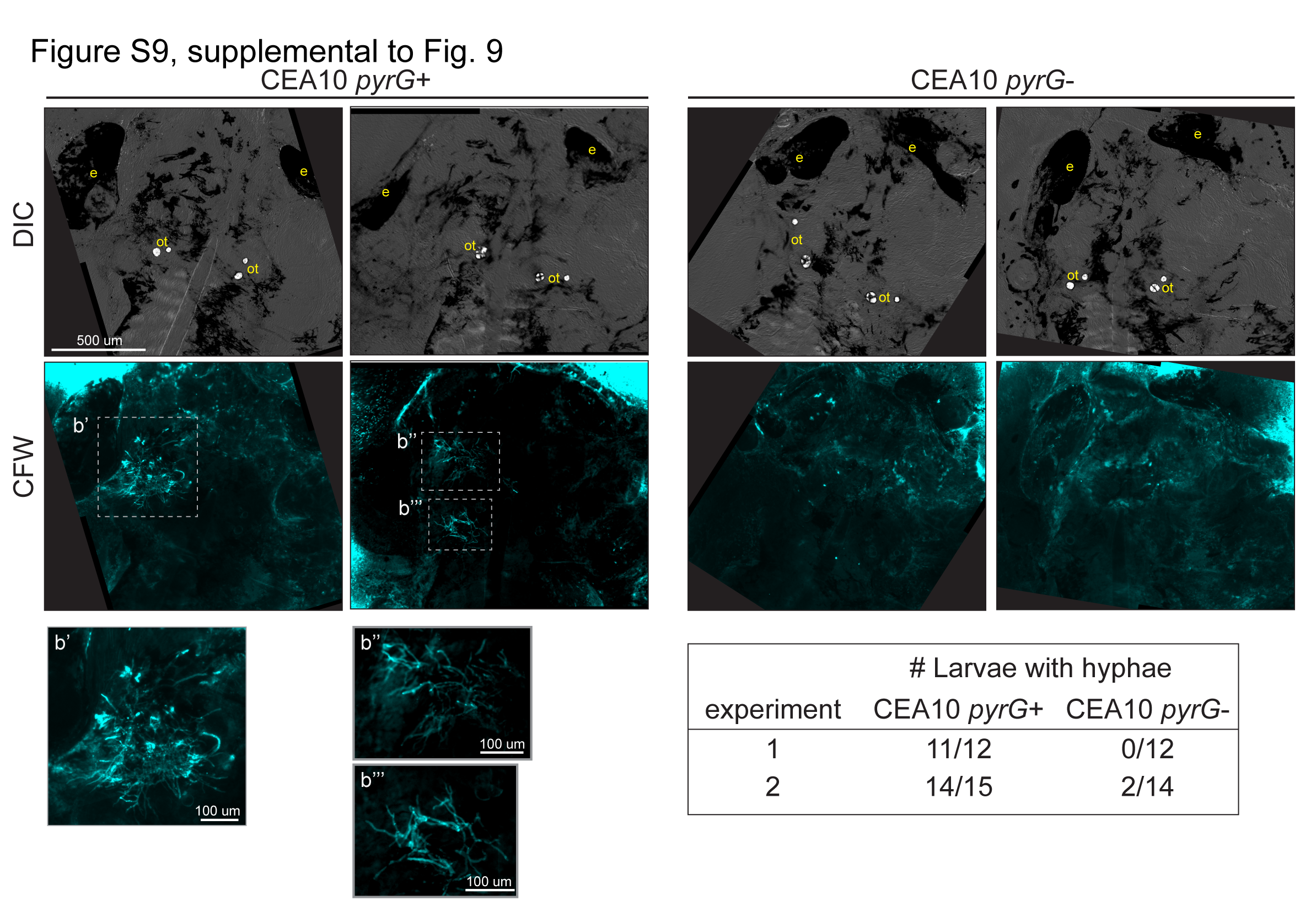

Supplement: S9 Fig — Phagocyte-deficient larvae (pu.1 morphant) were infected with non-fluorescent spores of CEA17 KU80Δ (CEA10 pyrG+) or CEA17 (CEA10 pyrG-). 1 dpi A. fumigatus growth was visualized in flattened larvae with calcofluor white (CFW) staining and representative widefield images are shown. e = eye, ot = otic vesicle. Number of larvae with hyphal growth in 2 replicates was quantified. (TIF) [file ppat.1007229.s009.tif]
